# Synthesis, characterization and cytotoxic evaluation of metal complexes derived from new *N*′-(2-cyanoacetyl)isonicotinohydrazide

**DOI:** 10.1038/s41598-025-07689-w

**Published:** 2025-06-27

**Authors:** Mohamed H. Abdel-Rhman, Ghada Samir, Nasser M. Hosny

**Affiliations:** 1 Chemistry Department, Faculty of Science, Mansoura University, Mansoura, Egypt; 2 Chemistry Department, Faculty of Science, Port Said University, P.O. Box 4252, Port Said, Egypt

**Keywords:** Isoniazid, Spectroscopic studies, Chelation ligand, DFT modeling, Cytotoxicity activity, Coordination chemistry, Inorganic chemistry, Density functional theory

## Abstract

The novel ligand (H_2_L), N’-(2-cyanoacetyl)isonicotinohydrazide, has been synthesized *via* reacting the isonicotinic hydrazide with 1-cyanoacetyl-3,5-dimethylpyrazole. The keto-form of the free ligand has been evoked from its spectral data. Based on elemental analyses and mass spectra, the ligand formed 1:1 (M: L) metal complexes with the acetate salts of Cu(II), Co(II), Ni(II) and Zn(II). The complexes’ spectral analyses revealed that the ligand behaved as a mononegative bidentate *via* the hydrazonyl N^1^ and deprotonated enolized acetyl oxygen. Moreover, the DFT quantum chemical calculations revealed that the ligand had higher HOMO and lower LUMO energies than metal complexes, implying an electron donating character. Furthermore, the in vitro anticancer activity against HepG2 and HCT-116 cell lines displayed that the ligand was more potent than doxorubicin against both cell lines, although the metal complexes displayed lower efficacy.

**Supplementary Information:**

The online version contains supplementary material available at 10.1038/s41598-025-07689-w.

## Introduction

Pyridine heterocycles have distinctive importance in nature because of their versatile role as a building-block in the production of substantial biologically active compounds *via* interaction with enzymes, proteins, and DNA^1–3^. For instance, several pyridine-containing medications have been clinically approved as anticancer drugs, i.e., Sorafenib, Regorafenib, Vismodegib and Crizotinib^4–6^. Alternatively, the hydrazide compounds showed noteworthy biological activeness like antibiotic, antifungal and antitumor agents^7,8^. As well, they displayed readily keto-enol tautomerization, which enriched their ability to chelate transition metal ions^9^. In 1952, isonicotinohydrazide (pyridine-4-carboxylic acid hydrazide, INH) had been introduced as the highly effective drug “isoniazid” that controlled tuberculosis (TB), and since, INH is the core of tuberculosis treatment programs^10–12^. Besides, isoniazid hydrazone analogues were utilized as insecticides, anticoagulants, anticancer, antioxidants, and plant growth regulators owing to their physiological and biological activities^13^. Otherwise, the cyanoacetyl hydrazides incorporate both electrophilic and nucleophilic centers, and thus used in the synthesis of many heterocyclic compounds^14^. Also, they were broadly engaged in pharmaceuticals as antibacterial and herbicides agents^15,16^.

In the 1960’s, the metal-based cancer therapy began with discovery of the cisplatin’s, a platinum(II) coordination complex, significant antitumor efficacy against various cancers^17,18^. The platinum-based drugs like cisplatin, carboplatin, and oxaliplatin efficacy resulted from forming DNA adducts, that interrupt DNA replication and transcription, leading to apoptosis^18^. However, the major challenge with platinum-base drugs was their toxicity. To overcome such obstacle, extensive examination of other metals-based anticancer agents had been performed^19,20^, where they presented varied mechanisms of action, such as targeting different cellular components, promoting production of reactive oxygen species (ROS) and manipulating enzyme activity^19–21^.

Moreover, transition metals, such as Cu(II), Co(II), Ni(II) and Zn(II), are vital for human, due to their participation in structures of metalloenzymes and cofactors of oxidative enzymes, as well as vitamins, which govern the nucleic acid replication, transcription and repair^22,23^. Such transition metals were particularly interesting for developing anticancer drugs exhibiting varied mechanisms of action, as they could bind to diverse bioactive ligands with several coordination numbers and oxidation states^24^.

For instance, the Cu(II) complexes exhibited efficacy by ROS generation inducing oxidative stress, proteasome inhibition, and direct DNA interaction, while the Co(II) complexes were selectively targeting oxygen-deprived tumor regions, inducing apoptosis *via* mitochondrial pathways. Moreover, the Ni(II) complexes had DNA-damaging capabilities and enzyme inhibition, whereas the Zn(II) complexes had disrupted cancer cell signaling pathways, induced apoptosis, inhibited metalloproteinases matrix, and were utilized in drug delivery systems to enhance therapeutic efficacy^19,20,24^. Eventually, the hepatocellular carcinoma (HepG2) and colorectal carcinoma (HCT-116) cancer cell lines have been utilized widely in in-vitro anticancer studies due to their substantial global impact, relevance to significant human malignancies and their well-characterized properties^25,26^.

Building on the well-established bioactivity of isonicotinic hydrazide and cyanoacetyl hydrazide derivatives, we designed a novel multifunctional hybrid, N’-(2-cyanoacetyl) isonicotinohydrazide, as versatile ligand that could form stable and bioactive metal complexes with Cu(II), Co(II), Ni(II), and Zn(II) transition metals, having vital biological roles and proven anticancer mechanisms along with variable coordination geometries. In addition to our earlier endeavors to develop new isonicotinic hydrazide conjugates and its metal complexes which may have antitumor activity^27–35^, the current study displays the synthesis, characterization and anticancer application versus HepG2 and HCT-116 cell lines of the novel ligand, N’-(2-cyanoacetyl)isonicotinohydrazide, and its transition metal complexes of copper(II), cobalt(II), nickel(II) and zinc(II).

## Experimental

### Materials and apparatuses

The isonicotinic hydrazide (99.0%), 1-cyanoacetyl-3,5-dimethylpyrazole (97%) and metal acetate salts (Cu(OAc)_2_.H_2_O, Co(OAc)_2_.4H_2_O, Ni(OAc)_2_.4H_2_O or Zn(OAc)_2_.2H_2_O) have been obtained as analytical grade from Aldrich or Merck. Fetal Bovine serum was bought from GIBCO, UK. Both tetrazolium bromide (MTT) and RPMI-1640 medium were brought from Sigma. The cell lines, hepatocellular (HepG2) and colon (HCT-116) carcinoma, were obtained from ATCC, Egypt.

The carbon, hydrogen and nitrogen contents were determined using CHN analyzer Perkin-Elmer model 2400, whereas the metals content were verified by standard methods^36^. The ThermoNicolet IS10 spectrometer have been utilized for recording the FT-IR spectra, as KBr discs. The Unicam UV/Vis UV2 spectrometer used to measure the electronic spectra. The Jeol Delta 2 spectrometer (500 MHz) have been used for measuring the ^1^H and ^13^C NMR spectra. The Brucker E 500 ESR spectrometer was utilized in obtaining the spectrum of Cu(II) complexes (480–6480 Gauss) at room temperature, 9.808 GHz, 100 kHz field modulation. The TGA behavior has been determined on a Schimadzu 50 instrument under N_2_-flow (10 cm^3^/min) and heating rate 15 ºC/min. ThermoScientific DSQ II spectrometer has been employed in recording the mass spectra. A Sherwood Scientific magnetic balance has been used for measuring the magnetic susceptibility of metal complexes.

### Synthesis of ligand and metal complexes

**Fig. 1:**
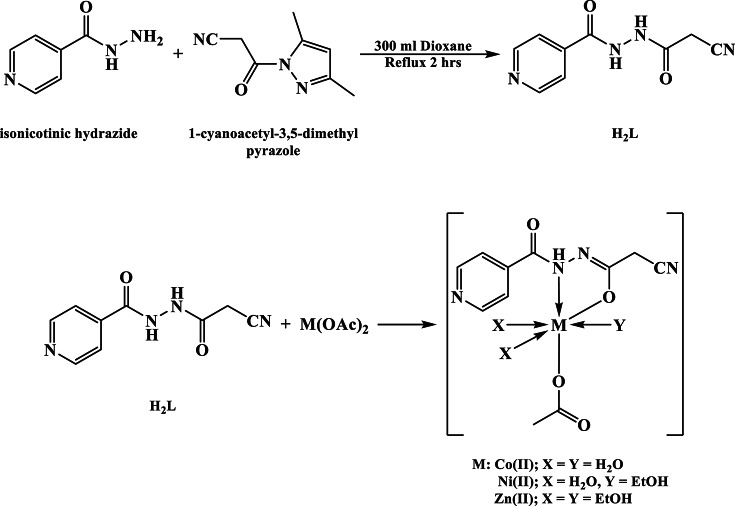
The synthesis of ligand and its complexes.

In dioxane, a mixture of isonicotinic hydrazide (0.01 mol) and 1-cyanoacetyl-3,5-dimethylpyrazole (0.01 mol) was refluxed for 2 h, where on cooling in ice, the N’-(2-cyanoacetyl)isonicotinohydrazide (H_2_L) was observed as pale-yellow precipitate^33^. It was filtered off, washed successfully with ethanol as well as distilled water, then dried in a vacuum desiccator over anhydrous CaCl_2_ (Fig. 1).

Yield = 89.0%, m. p. = 211 °C. MS *m/z* (%): 204.30 (6.97%). Anal. Calcd. for C_9_H_8_N_4_O_2_ (M. Wt. 204.19): C, 52.94; H, 3.95; N, 27.44%. Found: C, 53.21; H, 4.17; N, 27.52%.

To alcoholic solution of H_2_L (1.0 mmol, 0.204 g), an aqueous solution of Cu(II), Co(II), Ni(II) or Zn(II) acetate (1.0 mmol; 0.181, 0.172, 0.249 and 0.183 g, respectively) was poured dropwise with agitation. The admixture was heated under reflux for 2 h^33^. The solid particles filtered, washed with heated ethyl alcohol pursued by diethyl ether, and dried in a vacuum desiccator over anhydrous CaCl_2_ (Fig. 1).

[Cu(HL)(OAc)]EtOH: Yield = 80.1%, m. p. = > 300 °C. MS *m/z* (%): 371.41 (20.67%). Anal. Calcd. for C_13_H_16_CuN_4_O_5_ (M. Wt. 371.84): C, 41.99; H, 4.34; N, 15.07; M, 17.09%. Found: C, 41.54; H, 3.94; N, 15.27; M, 16.86%.

[Co(HL)(OAc)(H_2_O)_3_](H_2_O)_3_: Yield = 78.4%, m. p. = > 300 °C. MS *m/z* (%): 429.01 (21.67%). Anal. Calcd. for C_11_H_22_CoN_4_O_10_ (M. Wt. 429.25): C, 30.78; H, 5.17; N, 13.05; M, 13.73%. Found: C, 30.79; H, 5.31; N, 13.28; M, 13.62%.

[Ni(HL)(OAc)(EtOH)_2_(H_2_O)](H_2_O)_2_: Yield = 82.6%, m. p. = > 300 °C. MS *m/z* (%): 466.86 (20.51%). Anal. Calcd. for C_15_H_28_N_4_NiO_9_ (M. Wt. 467.10): C, 38.57; H, 6.04; N, 11.99; M, 12.57%. Found: C, 38.32; H, 5.86; N, 12.16; M, 12.74%.

[Zn(HL)(OAc)(EtOH)_3_]H_2_O: Yield = 88.2%, m. p. = > 300 °C. MS *m/z* (%): 483.69 (24.35%). Anal. Calcd. for C_17_H_30_N_4_O_8_Zn (M. Wt. 483.83): C, 42.20; H, 6.25; N, 11.58; M, 13.51%. Found: C, 42.40; H, 6.09; N, 11.91; M, 13.38%.

### Molecular modeling

Geometry optimization of the ligand and its complexes have been performed at density functional theory/Becke3-Lee-Yang-Parr exchange-correlation functional (DFT/B3LYP/6–311^++^G(d, p) basis set) level^37–39^ comprised in Gaussian 09 W^40^. The HOMO and LUMO (highest and lowest occupied molecular orbits) were displayed by GaussView program^41^.

### Cytotoxicity assay

The ligand and its metal complexes cytotoxicity assay were explored using the tetrazolium bromide (MTT) assay and doxorubicin as a standard anticancer drug. The cell lines were cultured in RPMI-1640 medium (10% fetal bovine serum). Antibiotics (100 units/mL penicillin and 100 µg/mL streptomycin) were added in a 5% CO_2_ at 37 °C incubator. The cells have been incubated with the investigated compounds for 24 h, then 20 µL of MTT solution (5 mg/mL) was incubated for 4 h. A 100 µL of DMSO (dimethyl sulfoxide) was added to the developed purple formazan, where at 570 nm, the absorbance was evaluated *via* plate reader (EXL 800, USA) and the relative cell viability% was estimated (A_570_ of treated samples/A_570_ of untreated sample)×100^42^.

## Results and discussion

The N’-(2-cyanoacetyl)isonicotinohydrazide (H_2_L) elemental analyses indicated that it had C_9_H_8_N_4_O_2_ formula, whereas its complexes were 1:1 (M: L) stoichiometry and were soluble only in DMF and DMSO. The complexes were non-electrolytic, where their molar conductivity in DMSO were 4.8–9.4 Ω^−1^cm^2^ mol^−1^^43^ (Table 1).

**Table 1:** Physical properties and elemental analyses data of H_2_L and its complexes.

Compound (Formula; M. Wt.)	Color	M.*P*. (^o^C)	Elemental analysis % Found (Calculated)			
			C %	H%	*N*%	M%
H_2_L (C_9_H_8_N_4_O_2_ = 204.19)	Yellow	211	53.21 (52.94)	4.17 (3.95)	27.52 (27.44)	-
[Cu(HL)(OAc)]EtOH (C_13_H_16_CuN_4_O_5_ = 371.84)	Green	> 300	41.54 (41.99)	3.94 (4.34)	15.27 (15.07)	16.86 (17.09)
[Co(HL)(OAc)(H_2_O)_3_](H_2_O)_3_ (C_11_H_22_CoN_4_O_10_ = 429.25)	Dark brown	> 300	30.79 (30.78)	5.31 (5.17)	13.28 (13.05)	13.62 (13.73)
[Ni(HL)(OAc)(EtOH)_2_(H_2_O)](H_2_O)_2_ (C_15_H_28_N_4_NiO_9_ = 467.10)	Dark green	> 300	38.32 (38.57)	5.86 (6.04)	12.16 (11.99)	12.74 (12.57)
[Zn(HL)(OAc)(EtOH)_3_]H_2_O (C_17_H_30_N_4_O_8_Zn = 483.83)	Off white	> 300	42.40 (42.20)	6.09 (6.25)	11.91 (11.58)	13.38 (13.51)

### Infrared spectra

**Fig. 2:**
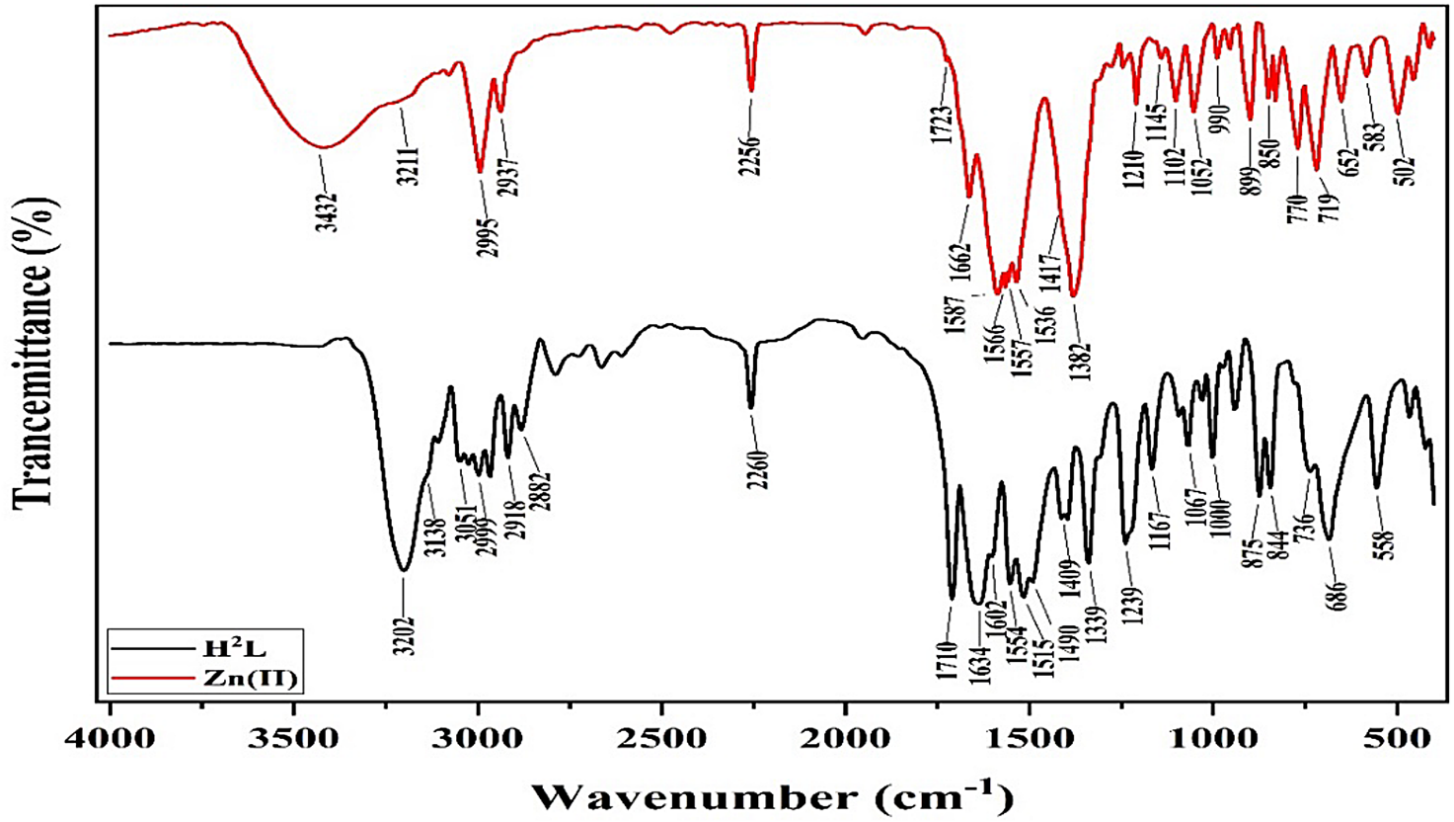
IR spectrum of H_2_L and [Zn(HL)(OAc)(EtOH)_3_]H_2_O complex.

**Fig. 3:**
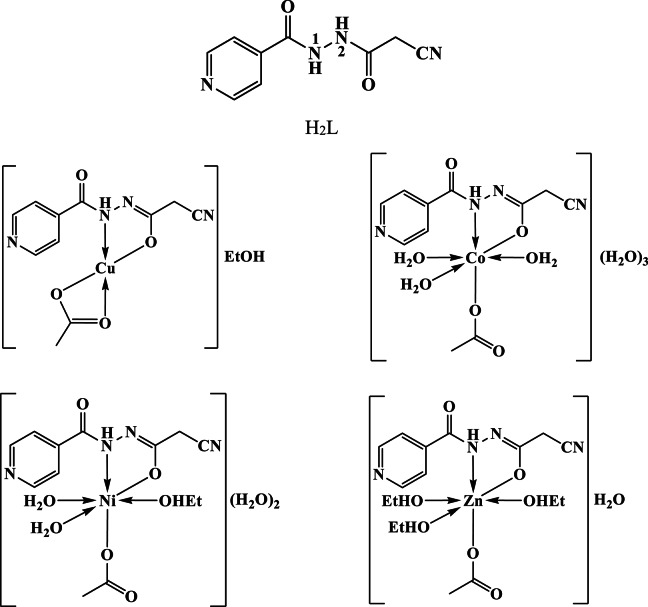
The suggested structures of the ligand and its metal complexes.

The IR spectrum of H_2_L presented a sharp band at 3202 cm^−1^ accompanied by a shoulder at 3138 cm^−1^, ascribed to ν(N^1^H) and ν(N^2^H)^29,44^, respectively. The band at 2260 cm^−1^ has been imputed to the ν(C ≡ N)^45^ (Fig. 2) (Table 2). As well, the presence of two bands at 1710 and 1645 cm^−1^ were designated to the acetyl and isonicotinoyl groups ν(C = O)^31,44^, respectively. Moreover, the bands at 1634 and 1602 cm^−1^ have been assigned to the ν(C = N)_Py_ and ν(C = C)_Py_^29^, however those at 1554 and 1515 cm^−1^ have been ascribed to amide II^46^, respectively. Thus, the ligand occurred in keto-form (Fig. 3).

Conversely, the IR spectra of metal complexes displayed a broad band with a shoulder at 3491 − 3430 and 3212–3204 cm^−1^, ascribed to the ν(OH)_solvent_^47^ and ν(N^1^H)^29,44^, respectively (Fig. 2 and S1). Further, the spectral data displayed a new band at 1650–1620 cm^−1^ owing to ν(C = N^2^)^29^, as well as, two bands at 1723 − 1717 and 1586–1392 cm^−1^ attributed to ν_as_(OAc) and ν_s_(OAc) of coordinated acetate ion in bidentate or monodentate mode (difference ≈ 135, 325–261 cm^−1^)^28,47^, respectively (Table 2).

Comparison of ligand and complexes spectral data implied that: (i) the disappearance of the ν(C = O)_acet_ and ν(N^2^H) bands accompanied by presence of a new one owing to ν(C = N^2^) designated that the enolization of such carbonyl group; (ii) the absence of ν(OH) band suggested that the newly formed hydroxyl group was deprotonated on binding the metal ion; (iii) the existence of the ν(C = O)_Py_ and ν(C ≡ N) vibration bands at comparable position discloses that the carbonyl oxygen atom did not coordinate with the metal ion^28,47^; (iv) the ν(N^1^H) shift to higher wavenumber designated its participation in chelating the metal ion^28,47^; and iiv) the new two bands, at 609 − 583 and 532–495 cm^−1^, were imputed to ν(M-O) and ν(M-N)^31,47^, respectively (Table 2).

As a result, the ligand existed as enol-keto configuration and coordinated to central metal as mononegative bidentate through the amide nitrogen and deprotonated enolized carbonyl oxygen group (Fig. 3).

**Table 2:** Infrared spectral data of the ligand and its complexes (cm^−1^).

Vibration	H_2_L	Complexes			
		[Cu(HL)(OAc)] EtOH	[Co(HL)(OAc) (H_2_O)_3_](H_2_O)_3_	[Ni(HL)(OAc)(EtOH)_2_ (H_2_O)](H_2_O)_2_	[Zn(HL)(OAc) (EtOH)_3_]H_2_O
ν(OH)_solvent_	-	3483	3491	3486	3432
ν(N^1^H)	3202	3208	3212	3204	3211
ν(N^2^H)	3138	-	-	-	-
ν_as_(OAc)	-	1721	1722	1717	1723
ν(C ≡ N)	2260	2258	2256	2254	2256
ν(C = O)_Actyl_	1710	-	-	-	-
ν(C = O)_Py_	1645	1680	1667	1671	1662
ν(C = N^2^)	-	1644	1620	1650	1620
ν(C = N)_Py_	1634	1644	1620	1616	1620
ν(C = C)_Py_	1602	1607	1600	1595	1601
ν_s_(OAc)	-	1586	1466	1392	1417
Amide II	1554, 1515	1560	1554	1558	1566
ν(N-N)	1167	1173	1163	1165	1145
ν(C-O)	1067	1063	1060	1064	1052
ρ(NH)	736,	727	705	700	719
ν(M-O)	-	607	609	600	583
ν(M-N)	-	515	532	495	502

### NMR spectra

**Fig. 4:**
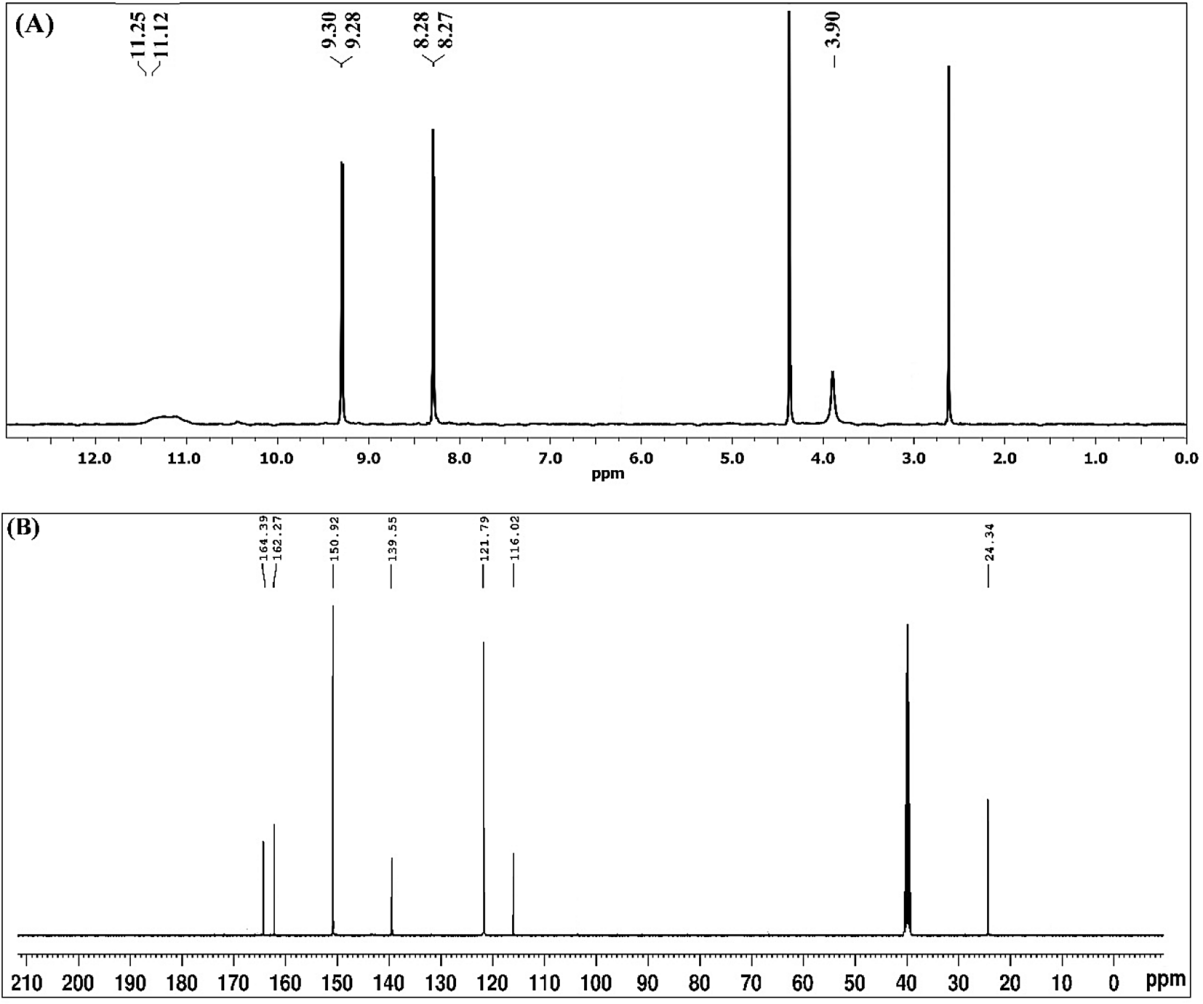
^1^H-NMR (A) and ^13^C-NMR (B) spectra of H_2_L in DMSO*-*d_6_.

The ^1^H-NMR spectrum of H_2_L in DMSO*-*d_6_ showed two singlet signals at 11.25 and 11.12 ppm, ascribed to the N^1^H and N^2^H protons^44,45^, respectively. In addition, the two doublet signals at 9.30 and 8.28 ppm were assigned to the pyridyl ring protons at 2,6- and 3,5-positions^28,45^, respectively (Fig. 4). While, the singlet signal presented at 3.90 ppm has been ascribed to the protons of methylene group (CH_2_)^44,45^. On addition of D_2_O, the downfield signals (> 11.0 ppm) were vanished, validating that they were corresponding to N^2^H and N^1^H protons, as well as the presence of the ligand in keto form.

Alternatively, the [Zn(HL)(OAc)(EtOH)_3_]H_2_O complex spectrum in DMSO*-*d_6_ displayed a singlet signal at 12.02 ppm, attributable to the N^1^H proton^44,45^. In addition, the spectrum presented two multiplet signals owing to pyridyl protons at 9.54 and 8.42 ppm^28,45^, respectively, while the singlet signal for the methylene (CH_2_) protons was at 4.19 ppm^44,45^. On comparison with the ligand’s spectrum, firstly, a new singlet signals at 2.10 ppm was attached to the CH_3_-protons of the acetate group^30^; secondly, the N^2^H signal was completely disappeared along with downfield shift of the N^1^H signal, which confirmed the enolization of the ligand and N^1^H participation in chelation to the metal ion.

On the other hand, the ^13^C-NMR spectrum of H_2_L in DMSO*-*d_6_ shows number of signals represent the number of chemically non-equivalent carbon atoms of the skeleton. The signals at 164.39 and 162.27 ppm were attributed to the carbonyl carbon of acetyl and isonicotinoyl groups^28,45^, respectively, while the signal at 116.02 ppm was assigned to the cyano’ s carbon^45^. Moreover, the pyridyl’s carbons at positions 2 and 6 were observed at 150.92 ppm and those at 3,5-positions were displayed at 121.79, whereas the carbon at 4-position was presented at 139.55 ppm^28,45^. The signal due to methylene (CH_2_) carbon was showed at 24.34 ppm^45^ (Fig. 4).

### Electronic spectra and magnetic moments

The ligand’s spectrum, in DMSO, demonstrated two bands at 216 and 310 nm ascribed to π→π^*^ transition of the pyridyl nucleus and carbonyl groups^45^, respectively. Further, the band at 389 nm with a shoulder at 429 nm have been designated to the n→π^*^ transitions of the pyridyl nucleus and carbonyl groups^45^, respectively (Figure S2).

The [Cu(HL)(OAc)]EtOH spectrum, in contrast to ligand, unveiled a new broad band at 678 nm with a shoulder at 787 nm owing to the ^2^B_1g_→^2^A_1g_ and ^2^E_g_→^2^T_2g_ transitions of square planar geometry^48,49^ (Figure S2). As well, the new band at 481 nm was attributed to the ligand to metal charge-transfer transition (LMCT)^48,49^. Furthermore, the intra-ligand transitions bands were observed at 218, 334 and 389 nm (Table 3). Besides, the complex had magnetic moment 1.87 B.M., which is within the usual range for Cu(II) complexes despite of stereochemistry, 1.75–2.20 B.M^49^.

Whereas, the [Co(HL)(OAc)(H_2_O)_3_](H_2_O)_3_ complex showed a new band at 524 nm with two shoulders at 631 and 729 nm, where the former have been attributed to LMCT, and the latter’s were assigned to the ^4^T_1g_(F)→^4^T_1g_(P) (υ_3_) and ^4^T_1g_(F)→^4^A_2g_(P) (υ_2_) transitions, respectively, signifying octahedral structure of the metal complex^48^ (Figure S2). The ligand field parameters, υ_1_, B and 10Dq were estimated utilizing the obtained spin-allowed transitions of the d^7^-system and were 6489, 680, and 7476 cm^−1^, respectively, which were in the octahedral structure range^27,48^. The Co(II) complex magnetic moment was 5.05 B.M., in agreement with the normal standards of octahedral geometry, 4.3–5.2 B.M^50^. (Table 3).

Moreover, the spectrum [Ni(HL)(OAc)(EtOH)_2_(H_2_O)](H_2_O)_2_ offered two bands at 475 and 529 nm beside a shoulder at 664 nm designated to LMCT, ^3^A_2g_(F)→^3^T_1g_(F) (ν_2_) and ^3^A_2g_(F)→^3^T_2g_(F) (ν_1_) transitions, respectively, of the Ni(II) ions with octahedral configuration^48^ (Figure S2). The d^8^-system ligand field parameters, B and 10Dq, in addition ν_3_ have been resolved utilizing the observed values of ν_2_ and ν_1_ and were B = 358, 10Dq = 15,030 and ν_3_ = 31,510 cm^−1^, validating the octahedral structure^27,48^. Furthermore, the complex’s magnetic moment was 3.26 B.M., matched with the normal standards of the octahedral geometry (2.9–3.3 B.M.)^48^ (Table 3).

Finally, the [Zn(HL)(OAc)(EtOH)_3_]H_2_O complex exhibited three bands at 215, 332 and 390 nm, alongside a shoulder at 429 nm ascribed to intra-ligand transitions π→π^*^and n→π^*^^45^, respectively, as well as, a new band at 458 nm that assigned to LMCT^48,49^ (Figure S2).

**Table 3:** Ligand and complexes electronic spectral bands and magnetic moment.

Compound	Band position (transition) (nm)	µ_eff_ (B.M.)
H_2_L	216 (π→π^*^)_Py_; 310 (π→π^*^)_C=O_; 389 (n→π^*^)_Py_; 429 (n→π^*^)_C=O_	-
[Cu(HL)(OAc)]EtOH	218 (π→π^*^)_Py_, 334 (π→π^*^)_C=O_; 389 (n→π^*^); 481 (LMCT); 678 (^2^B_1g_→^2^A_1g_); 787 (^2^E_g_→^2^T_2g_)	1.87
[Co(HL)(OAc)(H_2_O)_3_(H_2_O)_3_	219 (π→π^*^)_Py_; 333 (π→π^*^)_C=O_; 477 (n→π^*^); 524 (LMCT); 631 (^4^T_1g_(F)→^4^T_1g_(P) (υ_3_)); 729 (^4^T_1g_(F)→^4^A_2g_(P) (υ_2_))	5.05
[Ni(HL)(OAc)(EtOH)_2_ (H_2_O)](H_2_O)_2_	218 (π→π^*^)_Py_; 340 (π→π^*^)_C=O_; 397 (n→π^*^); 475 (LMCT); 529 (^3^A_2g_(F)→^3^T_1g_(F) (ν_2_)); 664 (^3^A_2g_(F)→^3^T_2g_(F) (ν_1_))	3.26
[Zn(HL)(OAc)(EtOH)_3_]H_2_O	215 (π→π^*^)_Py_; 332 (π→π^*^)_C=O_; 390, 429 (n→π^*^); 458 (LMCT)	-

### Mass spectra

**Fig. 5:**
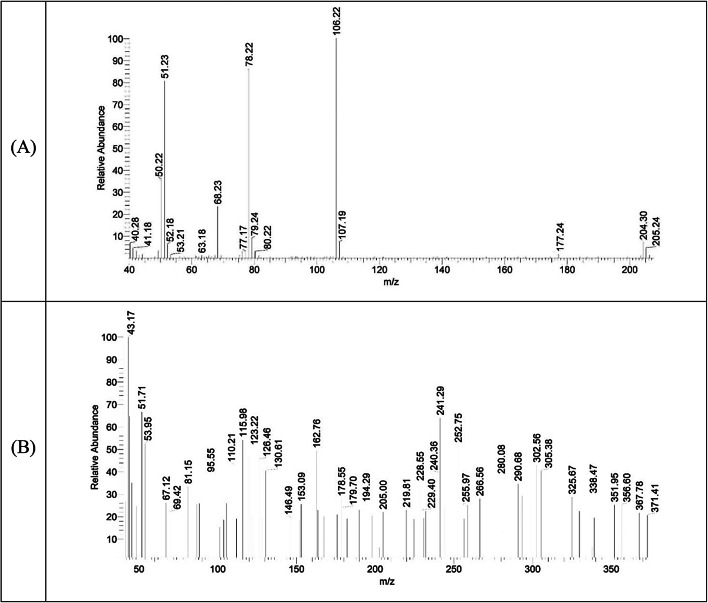
Mass spectra of H_2_L (A) and [Cu(HL)(OAc)]EtOH complex (B).

**Fig. 6:**
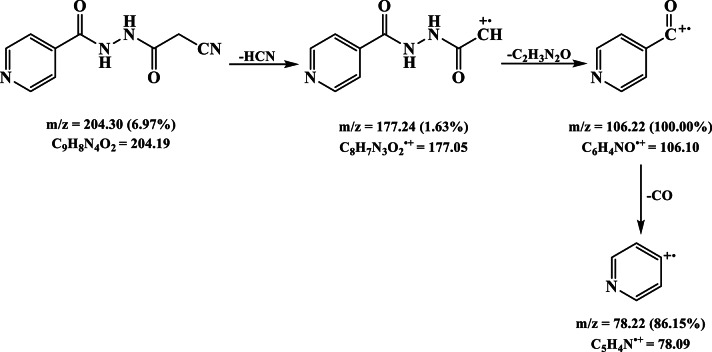
Suggested fragmentation pattern of the ligand.

The mass spectrum of the ligand exhibited molecular ion peak at *m/z* = 204.30 (6.97%) which matched with the proposed formula for the ligand (C_9_H_8_N_4_O_2_; M. Wt. 204.19) (Fig. 5). Furthermore, the spectrum presented additional peak at *m/z* = 177.24 (1.63%) that has been attributed to C_8_H_7_N_3_O_2_^•+^ radical (F. Wt. 177.05) resulted from the loss of HCN moiety. Additionally, the base peak was observed at 106.22 (100.00%) and assigned to the C_6_H_4_NO^2•+^ (F. Wt. 106.10) which underwent further fragmentation losing CO moiety to produce a new pyridyl radical ion C_5_H_4_N^•+^ (F. Wt. 78.09), *m/z* = 78.22 (86.15%) (Fig. 6).

**Fig. 7:**
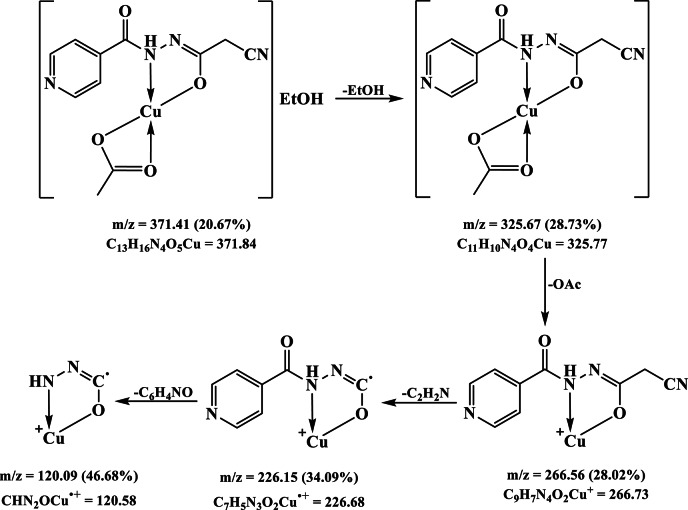
Proposed fragmentation pattern of the [Cu(HL)(OAc)]EtOH complex.

Furthermore, the [Cu(HL)(OAc)]EtOH mass spectrum unveiled a molecular ion peak at *m/z* = 371.41 (20.67%) which matched with the proposed formula C_13_H_16_CuN_4_O_5_ (Mol. Wt. 371.84) (Table 1). The peak at *m/z* = 325.67 (28.73%) has been ascribed to the loss of ethanol molecule presenting [Cu(HL)(OAc)] (F. Wt. 325.77), which underwent additional degradation by expelling acetate ion to give a peak at *m/z* = 266.56 (28.02%) corresponding to [Cu(HL)]^+^ formula (F. Wt. 266.73) (Fig. 5). Moreover, the latter moiety decomposed in two subsequent steps giving peaks at *m/z* = 226.15 (34.09%) and 120.09 (46.68%), which have been imputed to the fragments C_7_H_5_N_3_O_2_Cu^•+^ (F. Wt. 226.68) and CHN_2_OCu^•+^ (F. Wt. 120.58), respectively (Fig. 7). Likewise, the Co(II), Ni(II) and Zn(II) complexes exhibited more complicated spectra but showed molecular ion peak at *m/z* = 429.01 (21.67%), 466.86 (20.51%) and 483.69 (24.35%) (Figure S3), that matched with the proposed formulae, [Co(HL)(OAc)(H_2_O)_3_](H_2_O)_3_ (Mol. Wt. 429.25), [Ni(HL)(OAc)(EtOH)(H_2_O)_2_](EtOH)H_2_O (Mol. Wt. 467.10), and [Zn(HL)(OAc)(EtOH)_3_]H_2_O (Mol. Wt. 483.83), respectively (Table 1).

### ESR spectrum of Cu(II) complex

**Fig. 8:**
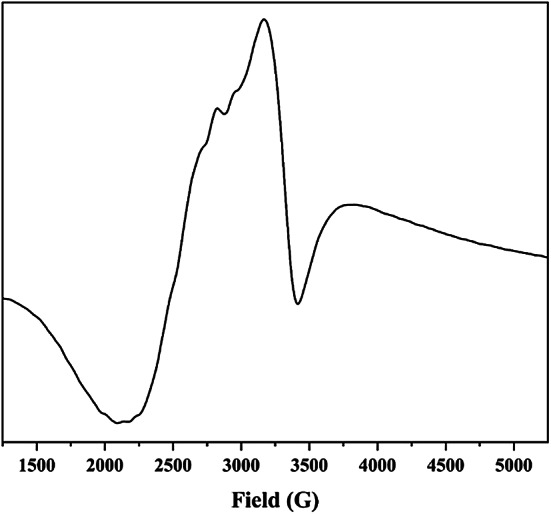
ESR spectra of [Cu(HL)(OAc)]EtOH complex.

The Cu(II) complexes with octahedral, square pyramidal or square planar geometries exhibited $\:{\text{d}}_{{\text{x}}^{2}-{\text{y}}^{2}}$ ground state. The ground term has been determined using the g-tensor values obtained from the ESR spectrum, where ^2^B_1g_ derived if g_||_ > g_⊥_ > 2.0023, while ^2^A_1g_ if g_⊥_ > g_||_ > 2.0023^51^. The [Cu(HL)(OAc)]EtOH complex’s spectrum in solid-state exhibited g-tensor values that followed the relation g_||_ (2.19) > g_⊥_ (2.09) > 2.0023. Therefore, the Cu(II) had a $\:{\text{d}}_{{\text{x}}^{2}-{\text{y}}^{2}}$ ground state typical for the octahedral or square planar geometry^49^ with significant covalent character of metal-ligand bonds as the g_||_ < 2.3^52^ (Fig. 8). The axial symmetry parameter, G = (g_||_−2)/(g_⊥_−2), was 2.07, suggesting significant copper-copper exchange interactions in the solid state as its less than 4^53^. Moreover, the g-tensors have been employed to estimate the magnetic moment (1.84 BM)^54^, which is in good agreement with the experimental value 1.87 B.M. As a measure of covalency, the orbital reduction factor (K) was calculated using the following simplified expressions^34,55^, where K = 1 for ionic, K < 1 for covalent environments, $\:{K}_{\parallel\:}$ and $\:{K}_{\perp\:}$ are the parallel and perpendicular components of orbital reduction factor, respectively (Table 4).

$$\:{\text{K}}_{\parallel\:}^{2}=\frac{\left({\text{g}}_{\parallel\:}-2.0023\right)}{8\times\:{{\uplambda\:}}_{^\circ\:}}\times\:\text{d}-\text{d}\:\text{t}\text{r}\text{a}\text{n}\text{s}\text{i}\text{t}\text{i}\text{o}\text{n}$$

$$\:{\text{K}}_{\perp\:}^{2}=\frac{\left({\text{g}}_{\perp\:}-2.0023\right)}{2\times\:{{\uplambda\:}}_{^\circ\:}}\times\:\text{d}-\text{d}\:\text{t}\text{r}\text{a}\text{n}\text{s}\text{i}\text{t}\text{i}\text{o}\text{n}$$

$$\:{\text{K}}^{2}=\frac{\left({\text{K}}_{\parallel\:}^{2}+2{\text{K}}_{\perp\:}^{2}\right)}{3}$$

The complex has K = 0.77, $\:{K}_{\parallel\:}$ = 0.64 and $\:{K}_{\perp\:}$= 0.83, indicating strong ionic character and in-plane π-bonding as $\:{K}_{\parallel\:}$<$\:{K}_{\perp\:}$.

**Table 4:** ESR calculated parameters of the Cu(II) complex.

Complex	g_||_	g_⊥_	G	K_||_	$\:{\varvec{K}}_{\perp\:}$	K
[Cu(HL)(OAc)]EtOH	2.19	2.09	2.07	0.64	0.83	0.77

### Thermal analyses

**Fig. 9:**
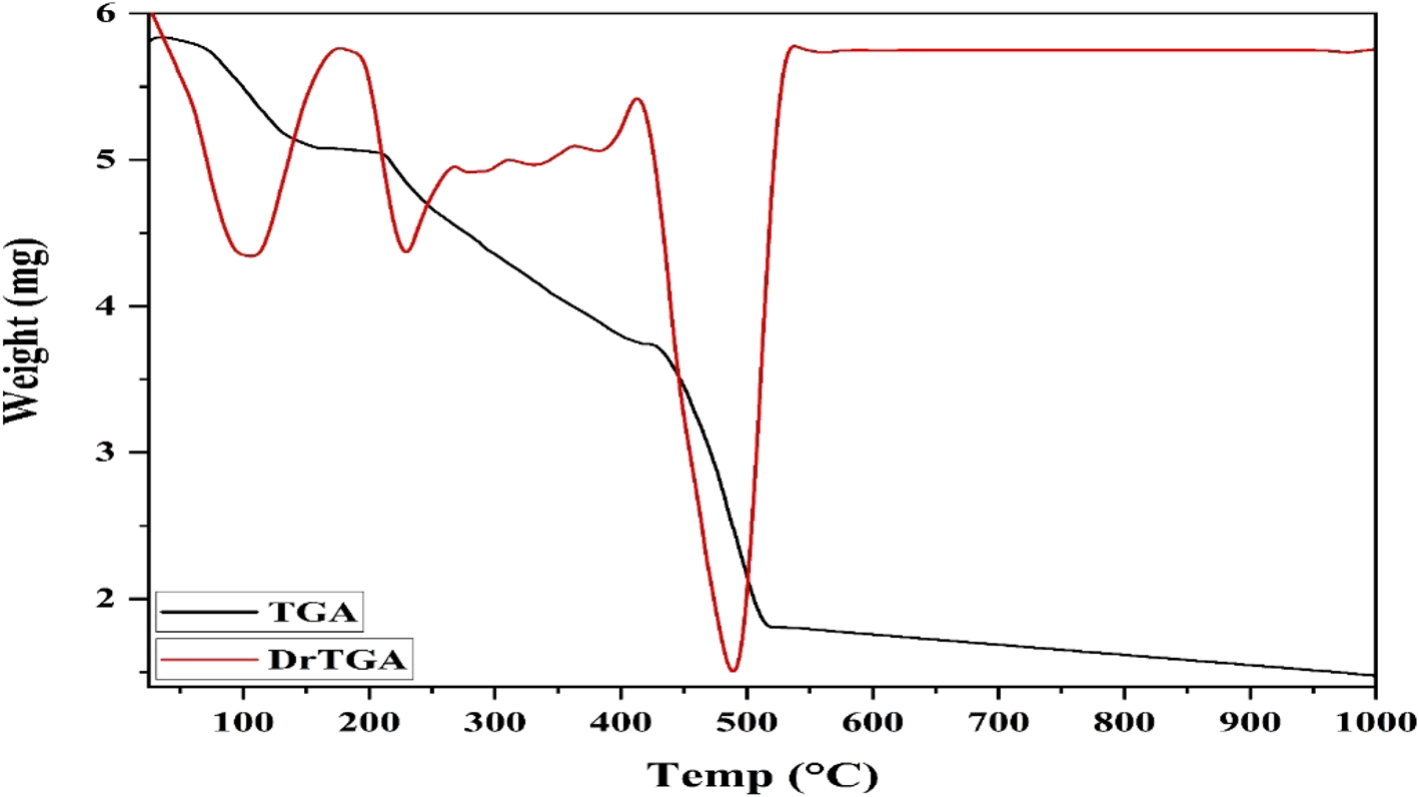
TGA of [Cu(HL)(OAc)]EtOH complex.

To investigate the thermal stability and endorse chemical structures of the yielded complexes, thermal gravimetric analysis (TGA) has been performed. The thermogram of [Cu(HL)(OAc)]EtOH demonstrated the decomposition stage at 25–160 °C, which was ascribed to the outside ethanol molecule exhausting (Found 12.45; Calcd. 12.39%). The second step was prolonged over 160–421 °C and imputed to the loss of coordinated acetate and cyanide anions (Found 22.95; Calcd. 22.88%) (Fig. 9). Further, the ligand decomposed in two stages 421–1000 °C, resulting in a residue of CuO (Found 21.73; Calcd. 21.39%) (Table 5).

The TG of [Co(HL)(OAc)(H_2_O)_3_](H_2_O)_3_ started decomposition stage at 25–175 °C region, that attributed to the elimination of water molecules outside the coordination sphere (Found 12.84; Calcd. 12.59%). The second step has been extended up to 408 °C and imputed to elimination of the coordinated water molecules and acetate anion in addition to both (C_5_H_4_N)CO and CH_2_CN fragments (Found 60.34; Calcd. 60.39%) (Figure S4). The final step was observed at 408–1000 °C leading to a residue of CoO (Found 18.03; Calcd. 17.46%) (Table 5).

Similarly, the [Ni(HL)(OAc)(EtOH)_2_(H_2_O)](H_2_O)_2_ complex’s thermogram presented the three degradation steps. The first at midpoint 105 °C has been attributed to the loss of the water molecules outside the coordination sphere (Found 8.07; Calcd. 7.72%). The second stage was extended over the 195–450 °C region and assigned to the loss of coordinated ethanol and water molecules accompanied with acetate anion and degradation of the ligand (Found 67.18; Calcd. 67.08%) (Table 5). While, the final stage was ascribed to loss of CHN_2_ fragment, leaving a metallic residue of NiO (Found 16.25; Calcd. 15.99%) (Figure S4).

Finally, the diagram of [Zn(HL)(OAc)(EtOH)_3_]H_2_O displayed the 1 st step at 25–128 °C, due to the loss of H_2_O molecule outside the coordination sphere (Found 3.56; Calcd. 3.72%) (Figure S4). The successive stages at 128–385 and 385–595 °C regions were assigned to loss of the coordinated ethanol molecules and acetate ion along with ligand fragments. The final step started at 595 ºC and led to a ZnO residue (Found 17.15; Calcd. 16.82%) (Table 5).

**Table 5:** Thermal gravimetric analyses data of the metal complexes.

Complex	Temp. range (°C)	Wt. loss (%)	Fragment (%)
[Cu(HL)(OAc)]EtOH	25–160	12.45	EtOH (12.39)
	160–421	22.95	OAc + CN (22.88)
	421–520	32.66	(C_5_H_4_N)CONH_2_ (32.84)
	520–1000	10.21	C_2_HN (6.50)
	Residue	21.73	CuO (21.39)
[Co(HL)(OAc)(H_2_O)_3_](H_2_O)_3_	25–175	12.84	3H_2_O (12.59)
	175–408	60.34	3H_2_O + OAc + (C_5_H_4_N)CO + CH_2_CN (60.39)
	408–1000	8.79	CHN_2_ (9.56)
	Residue	18.03	CoO (17.46)
[Ni(HL)(OAc)(EtOH)_2_(H_2_O)](H_2_O)_2_	25–195	8.07	2H_2_O (7.72)
	195–450	67.18	2EtOH + H_2_O + OAc + (C_5_H_4_N)CO + CH_2_CN (67.51)
	450–1000	8.50	CHN_2_ (8.78)
	Residual	16.25	NiO (15.99)
[Zn(HL)(OAc)(EtOH)_3_]H_2_O	25–128	3.56	H_2_O (3.72)
	128–385	9.80	EtOH (9.52)
	385–595	61.85	2EtOH + OAc + (C_5_H_4_N)CO + CH_2_CN (61.45)
	595–1000	7.64	CHN_2_ (8.49)
	Residue	17.15	ZnO (16.82)

### Molecular modeling

The DFT computations have been applied on ligand and its complexes to explore their geometrical structure, as well as the frontier molecular orbitals (FMO’s) shape and energy. The outcome geometrical characteristics, i.e., bond length, angle and dihedral angle, have been competed to those retrieved from x-ray single crystals of comparable compounds^56–58^, where trivial discrepancy was observed. Such discrepancies may be assigned to the absent of the intermolecular columbic interactions in the theoretical calculations since a single molecule in gaseous states is investigated, while in experimental, molecules are interacting in solid state crystal lattice^59^.

**Fig. 10:**
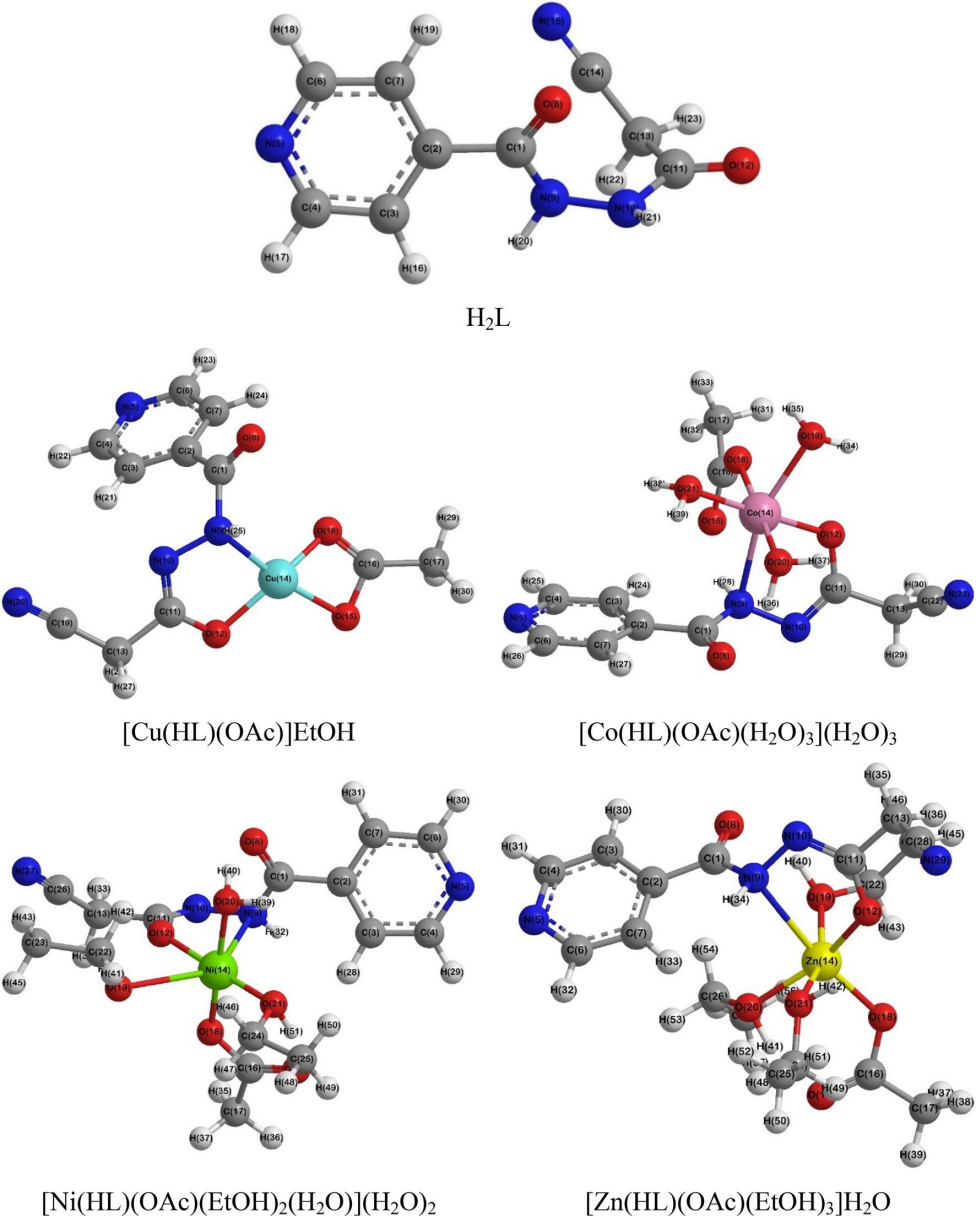
The DFT optimized structures of the H_2_L and its complexes (Created *via* Gaussian software version 09 W).

As shown in Fig. 10, the ligand’s DFT optimized structure revealed an angular configuration, in which isonicotinoyl carbonyl oxygen and hydrazonyl nitrogen atoms were positioned out the pyridyl’s plane (Table S1). Additionally, the optimized structure of the metal complexes revealed a square planar geometry for [Cu(HL)(OAc)]EtOH complex, while the [Co(HL)(OAc)(H_2_O)_3_](H_2_O)_3_, [Ni(HL)(OAc)(EtOH)_2_(H_2_O)](H_2_O)_2_ and [Zn(HL)(OAc)(EtOH)_3_]H_2_O complexes had octahedral structures consistent with the before-mentioned geometries. The geometrical properties of the ligand and complexes disclosed that:

i) The free ligand has CO_(Act)_-OC_(Act)_ = 1.22 Å and N^2^-CO_(Act)_ = 1.39 Å (keto-form), whereas in complexes, it was transformed to enol-form and so the former was elongated to 1.29–1.30 Å, whereas the latter was shortened to 1.30–1.31 Å, respectively.
ii) All the metal complexes exhibited deformation in bond lengths, namely, the M-N^1^H bond had 2.08–2.68 Å length, which lengthier than both of M-OC_(Act)_ and M-OAc (1.87–2.01 Å).
iii) Moreover, alternative deformation in complexes geometries was found in the bond angle data. For instance, the square planar [Cu(HL)(OAc)]EtOH complex has OC_(Act)_-Cu-N^1^H = 81.5° and O^1^_(OAc)_-Cu-N^1^H = 171.0°, which obviously diverged from the standard values, 90° and 180°, respectively. Also, the octahedral complexes unveiled angle deformation, where OC_(Act)_-M-N^1^H = 64.1–75.2° and O^1^_(OAc)_-Cu-N^1^H = 81.1–171.2° (Table S2-S3).

**Fig. 11:**
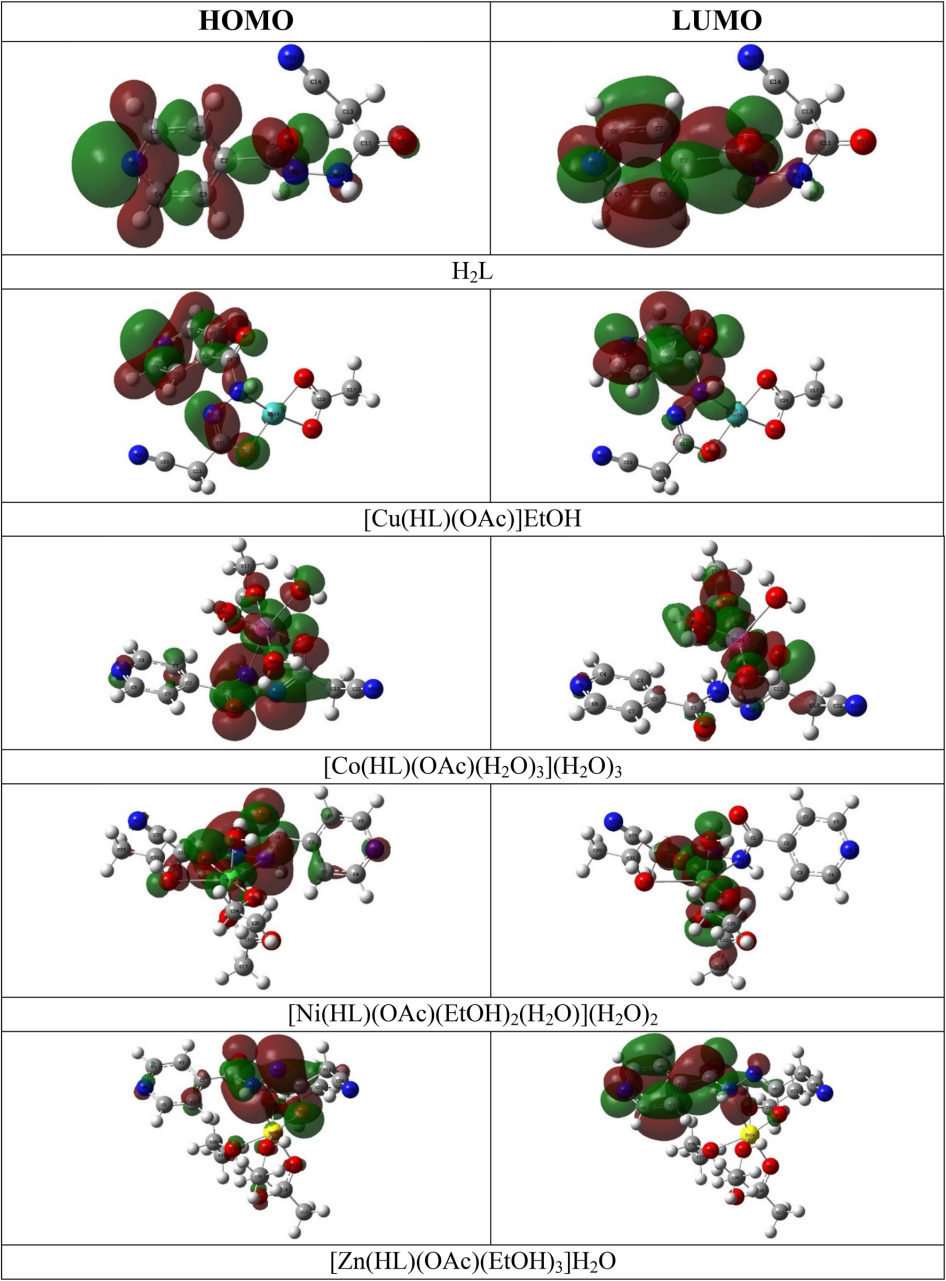
The 3D plots of HOMO and LUMO of the H_2_L and its complexes (Created *via* GaussView software version 5).

In addition, the highest occupied (HOMO) and lowest unoccupied (LUMO) molecular orbitals (FMO’s) influenced the molecular interactions, since they act as electron donor and acceptor, respectively. The LUMO-HOMO energy gap indicates the molecule’s chemical activity, as a molecule with a small gap is soft, more polar, chemically reactive and less kinetically stable^60^. As well, the little HOMO-LUMO interval signifies easy intramolecular charge-transfer^59^. The ligand FMO’s plots displayed that the HOMO was chiefly incorporated the π-orbitals of isonicotinoyl hydrazonyl moiety with the non-bonding orbits contain the oxygen and nitrogen lone pairs of electrons, while the LUMO was built of the isonicotinoyl hydrazonyl moiety π*-orbitals. So, the HOMO-LUMO charge-transfer may be designated as π→π^*^ and n→π^*^) transitions. The [Cu(HL)(OAc)]EtOH complex exhibited FMO’s configuration that almost matched to the ligand with considerable participation of the metal. However, the other complexes have almost similar configurations in which the metal and coordinated donor atoms have major contribution. So, the complexes HOMO-LUMO charge-transfer may be illustrated as LMCT and d→d transitions (Fig. 11).

According to the HOMO-LUMO energies, the ligand possessed the maximum HOMO energy, E_H_ = −7.66 eV, and lowest LUMO, E_L_ = −2.26 eV, revealing its electron donation character, while the metal complexes displayed lower E_H_ values, −6.62 - −7.53 eV, and higher E_L_, −2.31 - −3.14 eV. The ligand HOMO-LUMO gap, ΔE_H−L_, was the uppermost value, 5.40 eV, while, [Ni(HL)(OAc)(EtOH)_2_(H_2_O)](H_2_O)_2_ complex has the lowermost, 3.60 eV, and may be arranged as H_2_L > [Cu(HL)(OAc)]EtOH > [Zn(HL)(OAc)(EtOH)_3_]H_2_O > [Co(HL)(OAc)(H_2_O)_3_](H_2_O)_3_ > [Ni(HL)(OAc)(EtOH)_2_(H_2_O)](H_2_O)_2_ (Table 6).

Also, the FMO’s energies were utilized in estimating of some chemical reactivity descriptors, like electronegativity (χ), global hardness (*η*) and softness (*δ*). Besides, the electrophilicity (*ω*) along with the electron donating (ω^+^) and acceptance (ω^−^) powers, to assess the energy reduction owing to HOMO-LUMO electron flow and the ability to donate and accept electrons have been computed as follows^61,62^:

$$\:\chi\:=-\frac{1}{2}\left({E}_{HOMO}+{E}_{LUMO}\right)\:\eta\:=-\frac{1}{2}\left({E}_{HOMO}-{E}_{LUMO}\right)\:\delta\:=\frac{1}{\eta\:}$$

$$\:\omega\:=\frac{{\chi\:}^{2}}{8\eta\:}\:{\omega\:}^{-}=\frac{{\left(3I+A\right)}^{2}}{16(I-A)}\:{\omega\:}^{+}=\frac{{\left(I+3A\right)}^{2}}{16(I-A)}$$

Table 6 showed that the ligand offered the maximum hardness, *η* = 2.70 eV, while the [Ni(HL)(OAc)(EtOH)_2_(H_2_O)](H_2_O)_2_ complex had the minimum, 1.80 eV. The softness displayed reversed order, indicating that the ligand has robust propensity to donate electrons. The molecules having electrophilicity index (ω) > 1.5 eV were classified as strong electrophile^61,63^. Thus, the examined compounds were robust electrophile, ω = 4.56–6.78 eV, following the order [Ni(HL)(OAc)(EtOH)_2_(H_2_O)](H_2_O)_2_ > [Co(HL)(OAc)(H_2_O)_3_](H_2_O)_3_ > [Cu(HL)(OAc)]EtOH > [Zn(HL)(OAc)(EtOH)_3_]H_2_O > H_2_L. Likewise, the electron donating (ω^+^) and acceptance (ω^−^) data indicated that they have more tendency to donate than receiving, where smaller values signify enhanced transaction^61,63^.

**Table 6:** The fmo’s energies and chemical reactivity descriptors of the ligand and its complexes (eV).

Compound	E_H_	E_L_	ΔE_H−L_	χ	η	δ	ω	ω^+^	ω^−^
H_2_L	−7.66	−2.26	5.40	4.96	2.70	0.37	4.56	2.42	7.38
[Cu(HL)(OAc)]EtOH	−7.53	−2.50	5.03	5.02	2.51	0.40	5.00	2.81	7.83
[Co(HL)(OAc)(H_2_O)_3_](H_2_O)_3_	−6.62	−2.64	3.98	4.63	1.99	0.50	5.39	3.33	7.96
[Ni(HL)(OAc)(EtOH)_2_(H_2_O)](H_2_O)_2_	−6.74	−3.14	3.60	4.94	1.80	0.56	6.78	4.54	9.48
[Zn(HL)(OAc)(EtOH)_3_]H_2_O	−6.92	−2.31	4.61	4.61	2.30	0.43	4.62	2.60	7.22

Eventually, transition metal complexes have been widely employed in production of non-linear optical material (NLO), which were used in several fields, like computing, image processing, and optical communication, because of to their structural variety, notable stability and variable electronic features^64,65^. The material’s NLO characteristics manipulated primarily through their dipole moment (µ), polarizability (α_total_), and first-order hyperpolarizabilities (β_total_)^66,67^. Moreover, the polarizability, as a molecular signifier, performed substantial function in the biological activeness^68^.

$$\:{\upmu\:}=({{\upmu\:}}_{\text{x}}^{2}+{{\upmu\:}}_{\text{y}}^{2}+{{\upmu\:}}_{\text{z}}^{2})\:{{\upalpha\:}}_{\text{t}\text{o}\text{t}\text{a}\text{l}}=\frac{({{\upalpha\:}}_{\text{x}\text{x}}+{{\upalpha\:}}_{\text{y}\text{y}}+{{\upalpha\:}}_{\text{z}\text{z}})}{3}$$

$$\:{{\upbeta\:}}_{\text{t}\text{o}\text{t}\text{a}\text{l}}=\sqrt{{\left({{\upbeta\:}}_{\text{x}\text{x}\text{x}}+{{\upbeta\:}}_{\text{x}\text{y}\text{y}}+{{\upbeta\:}}_{\text{x}\text{z}\text{z}}\right)}^{2}+{\left({{\upbeta\:}}_{\text{y}\text{y}\text{y}}+{{\upbeta\:}}_{\text{y}\text{z}\text{z}}+{{\upbeta\:}}_{\text{y}\text{x}\text{x}}\right)}^{2}+{\left({{\upbeta\:}}_{\text{z}\text{z}\text{z}}+{{\upbeta\:}}_{\text{z}\text{x}\text{x}}+{{\upbeta\:}}_{\text{z}\text{y}\text{y}}\right)}^{2}}$$

The data demonstrated that the [Cu(HL)(OAc)]EtOH complex has the highest dipole moment, 5.54 D, while the ligand appeared in the second place, 5.34 D. Compared to urea, all compounds have greater dipole moment, 2.83–4.03 times (Table 7). Moreover, the estimated total polarizability unveiled that the ligand has lesser standards than the metal complexes, α_total_ = 1.35 × 10^−23^ esu, where the complexes may be arranged as [Zn(HL)(OAc)(EtOH)_3_]H_2_O > [Ni(HL)(OAc)(EtOH)_2_(H_2_O)](H_2_O)_2_ > [Co(HL)(OAc)(H_2_O)_3_](H_2_O)_3_ > [Cu(HL)(OAc)]EtOH. Similarly, the first-order hyperpolarizability (β_total_) data indicated that the ligand has bottommost (0.39 × 10^−30^ esu), whilst the [Cu(HL)(OAc)]EtOH has the uppermost value (3.29 × 10^−30^ esu). Compared to urea, all the explored compounds unveiled developed standards than urea, 1.03–8.80 times^69^ (Table 7). So, the abovementioned outcomes led to that the metal complexes have improved polarizability and hyperpolarizability than the ligand, caused by their more ease intramolecular charge-transfer^64^.

**Table 7:** The computed dipole moment (µ), polarizability (α_total_), polarizability anisotropy (Δα) and first-order hyperpolarizability (β_total_) of investigated compounds.

Compound	µ (Debye)	µ/µ_urea_	α_total_ (esu×10^−23^)	Δα (esu×10^−24^)	β_total_ (esu×10^−30^)	β_total_/β_urea_
H_2_L	5.34	3.89	1.35	3.30	0.39	1.03
[Cu(HL)(OAc)]EtOH	5.54	4.03	1.88	1.83	3.29	8.80
[Co(HL)(OAc)(H_2_ O)_3_](H_2_O)_3_	4.17	3.04	2.19	4.82	1.54	4.13
[Ni(HL)(OAc)(EtOH)_2_ (H_2_O)](H_2_ O)_2_	4.32	3.14	2.55	6.01	1.45	3.89
[Zn(HL)(OAc)(EtOH)_3_]H_2_O	3.89	2.83	2.78	6.33	2.38	6.35

### Anticancer activity

Since discovery of *cis*-platin’s anticancer activity^17^, several mechanisms have been suggested to explain metal complexes’ efficacy in deterring cancer cells spread^70^, which resulted mainly from overproduction of ROS, fragmentation of DNA, collapse of mitochondrial membrane potential^71^. For instance, the anticancer activity of the Co(II) and Zn(II) complexes was attributed to cleavage and/or interacting with DNA *via* intercalation and groove binding DNA^72,73^. However, the mechanistic investigations on the antitumor action of Cu(II) and Ni(II) complexes revealed that they have strong DNA binding ability and inducing oxidative damage caused by ROS^74^.

The ligand and complexes in vitro cytotoxic efficacy were assessed against liver and colon carcinoma cell lines, HepG2 and HCT-116, respectively, *via* the MTT procedure and doxorubicin as a reference. The data disclosed that the ligand, IC_50_ = 3.87 ± 0.2 and 4.93 ± 0.3 µM, respectively, was more effective against both cell lines than some anticancer agents, i.e., doxorubicin (IC_50_ = 4.50 ± 0.2 and 5.23 ± 0.3), 5-Fluorouracil (5-FU) (IC_50_ = 12.92 ± 0.1 and 26.98 ± 1.9)^75,76^ and *cis*-platin (IC_50_ = 6.50 ± 0.4 and 8.51 ± 0.3)^75,76^ (Table 8 and S5). Likewise, the ligand exhibited higher activity than several isonicotinoyl hydrazide derivatives, which displayed IC_50_ = 6.59–30.72 and 7.68–35.4 µM against HepG2 and HCT-116, respectively^28–31,33–35^ (Table S5).

**Fig. 12:**
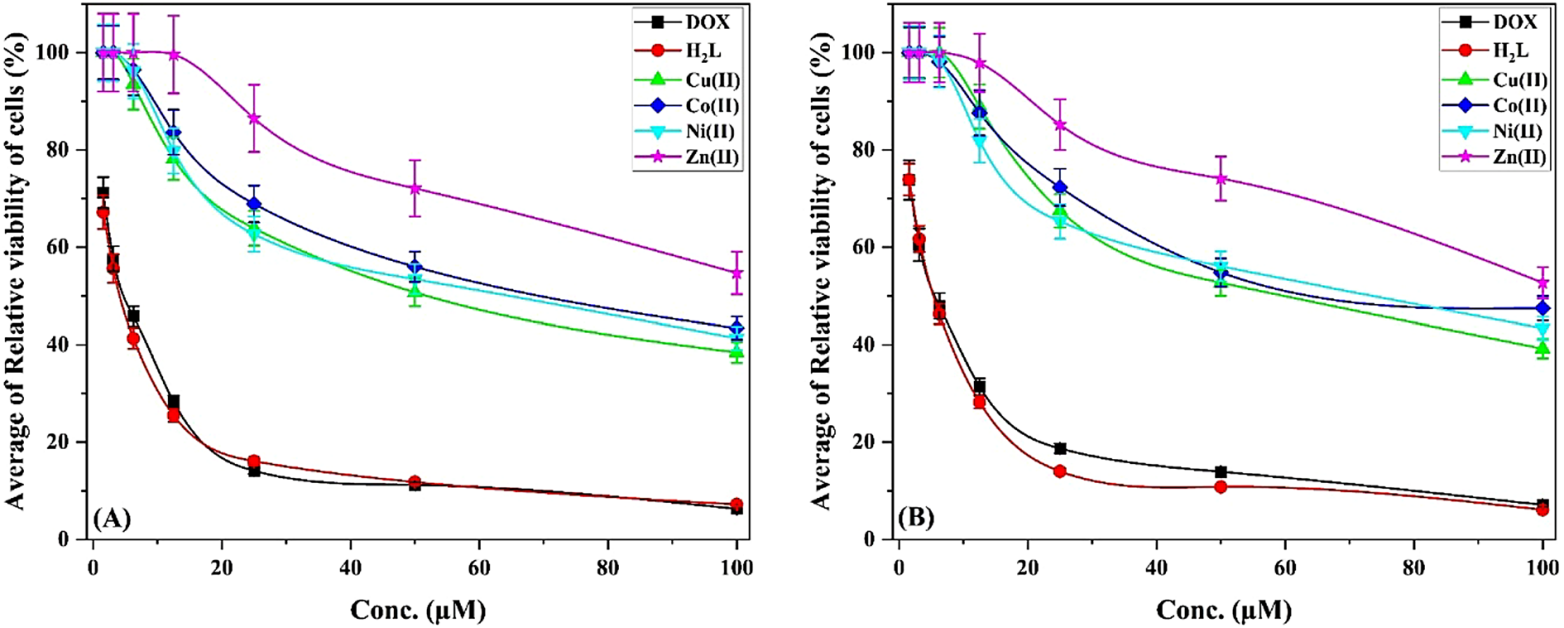
The Average of Relative viability of cells (%) of the H_2_L and complexes compared with the standard doxorubicin against HepG2 (A) and HCT-116 (B).

On contrary, despite their weak effect, the metal complexes demonstrated more strong influence against the HepG2 (IC_50_ = 53.24 ± 3.0–67.29 ± 3.7 µM) than HCT-116 (IC_50_ = 59.20 ± 3.2–74.12 ± 3.9 µM), where the [Cu(HL)(OAc)]EtOH was more effective than [Ni(HL)(OAc)(EtOH)_2_(H_2_O)](H_2_O)_2_, [Co(HL)(OAc)(H_2_O)_3_](H_2_O)_3_ and [Zn(HL)(OAc)(EtOH)_3_]H_2_O, respectively (Fig. 12). Generally, the current metal complexes displayed comparable activity to the previously reported for isonicotinoyl hydrazide derivatives complexes^28–31,33–35^ (Table S5).

The activity reduction of metal complexes compared to the ligand may be attributed to: (i) involvement of ligand’s active sites, that interact with the target receptor, in chelating the metal ion; (ii) change in ligand’s conformation associated with the complex formation, which may reduce its binding affinity; and/or (iii) difficulty in passing through cell membranes of the complexes, that were more hydrophilic, which reducing its effective concentration at the target site^77–81^.

**Table 8:** The in vitro cytotoxicity IC_50_ (µM) values of ligand and complexes (very strong: 1–10, strong: 11–20, moderate: 21–50, weak: 51–100 and non-cytotoxic: >100).

Compound	In vitro Cytotoxicity IC_50_ (µM)	
	HepG2	HCT-116
H_2_L	3.87 ± 0.2	4.93 ± 0.3
[Cu(HL)(OAc)]EtOH	53.24 ± 3.0	59.20 ± 3.2
[Co(HL)(OAc)(H_2_O)_3_](H_2_O)_3_	67.29 ± 3.7	74.12 ± 3.9
[Ni(HL)(OAc)(EtOH)_2_ (H_2_O)](H_2_O)_2_	58.31 ± 3.3	65.24 ± 3.6
[Zn(HL)(OAc)(EtOH)_3_]H_2_O	> 100	> 100
Doxorubicin	4.50 ± 0.2	5.23 ± 0.3

## Conclusion

The novel ligand, N’-(2-cyanoacetyl)isonicotinohydrazide (H_2_L), and its Cu(II), Co(II), Ni(II) and Zn(II) complexes have been synthesized and characterized. The ligand coordinated to the metal ion in a mononegative bidentate fashion, where the isolated complexes have octahedral geometry, except the [Cu(HL)(OAc)]EtOH complex, which is square planar. The DFT results showed a twisted configuration for the ligand, meanwhile, the metal complexes were coincided with the suggested geometrical structures. Moreover, the ligand has higher HOMO-LUMO energy gap than complexes following the order: H_2_L > [Cu(HL)(OAc)]EtOH > [Zn(HL)(OAc)(EtOH)_3_]H_2_O > [Co(HL)(OAc)(H_2_O)_3_](H_2_O)_3_ > [Ni(HL)(OAc)(EtOH)_2_(H_2_O)](H_2_O)_2_. Despite the in vitro potent activity of the ligand, the metal chelates exhibited weak effect against HepG2 and HCT-116 cell lines. The data disclosed that the ligand has higher efficacy against both cell lines than well-known anticancer agents, such doxorubicin, 5-Fluorouracil (5-FU) and *cis*-platin, as well as several isonicotinoyl hydrazide derivatives. While, its metal complexes displayed more potent effect against the HepG2 than HCT-116, which were comparable to those of isonicotinoyl hydrazide derivatives complexes (previously reported).

## Electronic supplementary material

Below is the link to the electronic supplementary material.

Supplementary Material 1

## Data Availability

All data generated or analyzed during this study are included in this published article [and its supplementary information files].
