# Supplementary material for: Synthesis, characterization and cytotoxic evaluation of metal complexes derived from new N′-(2-cyanoacetyl)isonicotinohydrazide
Source: Sci Rep. 2025 Jun 27;15:20335. doi: 10.1038/s41598-025-07689-w (PMC12205053; doi:10.1038/s41598-025-07689-w)
Supplement: Supplementary file 1 — Supplementary Material 1 [file 41598_2025_7689_MOESM1_ESM.docx]

**Synthesis, characterization and cytotoxic evaluation of 3d-metal complexes of the New N'-(2-cyanoacetyl)isonicotinohydrazide against HepG2 and HCT-116  cancer cell lines**

***Mohamed H. Abdel-Rhman^*a^, Ghada Samir^b^ and Nasser M. Hosny^*b^***

*^a^ Chemistry Department, Faculty of Science, Mansoura University, Mansoura, Egypt.
^b^ Chemistry Department, Faculty of Science, Port Said University, P.O. Box 4252, Port Said, Egypt.*

**
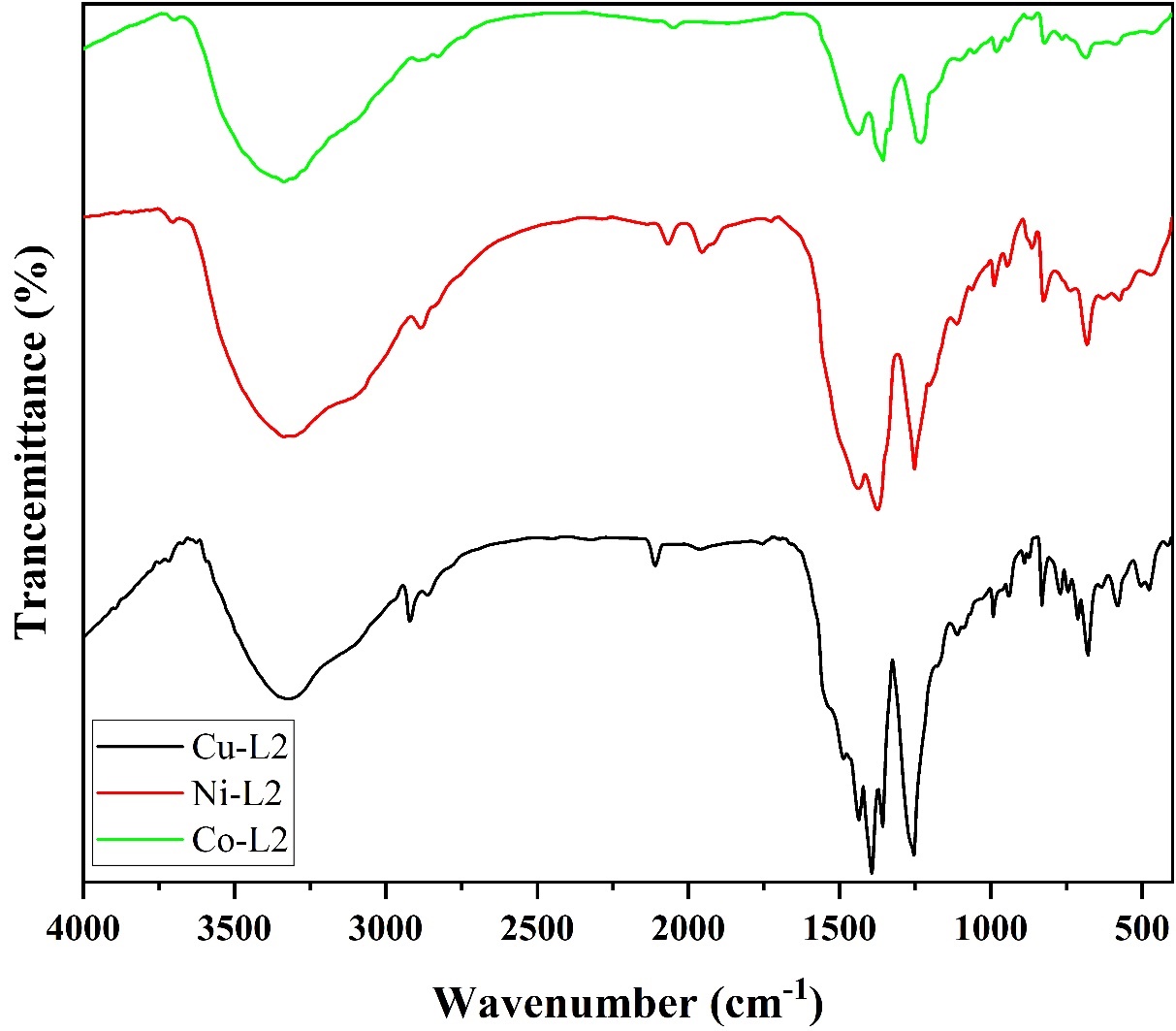
**

**Figure S1**. IR spectra of Cu(II), Co(II) and Ni(II) complexes

| **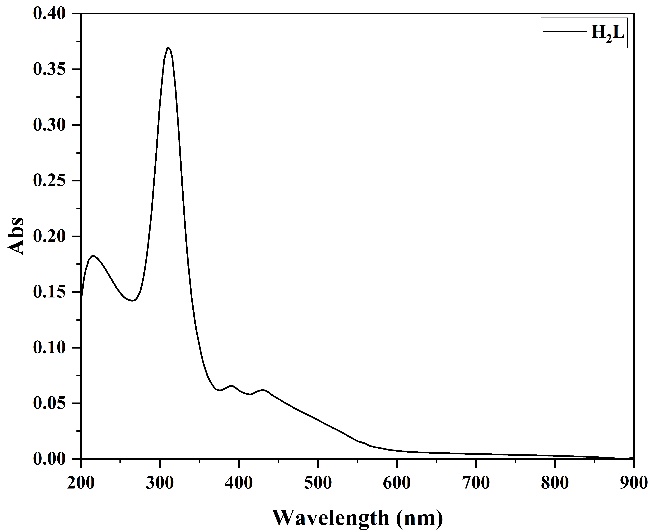** | **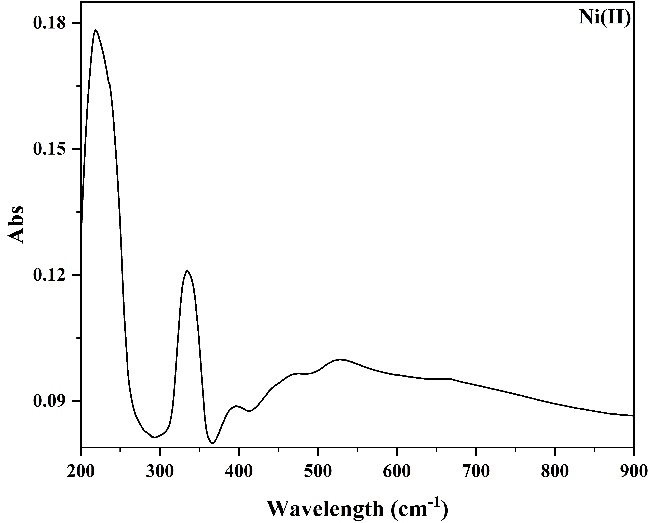** |
| --- | --- |
| **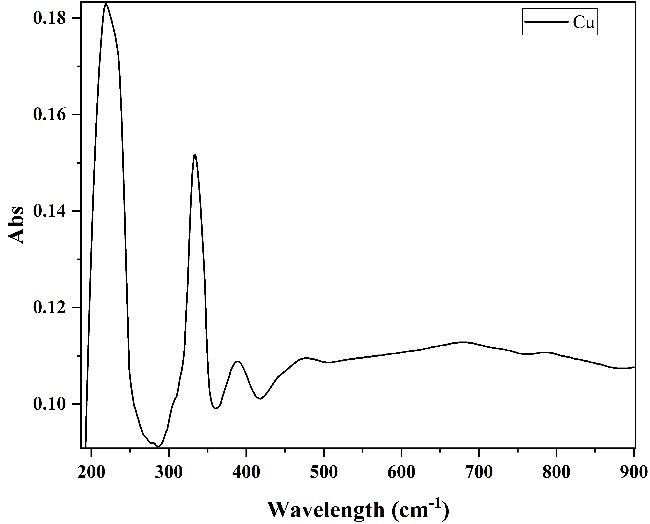** | **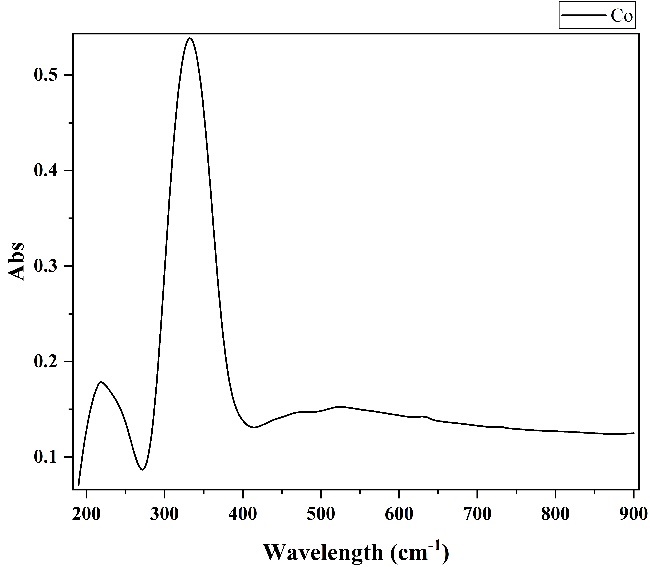** |
| **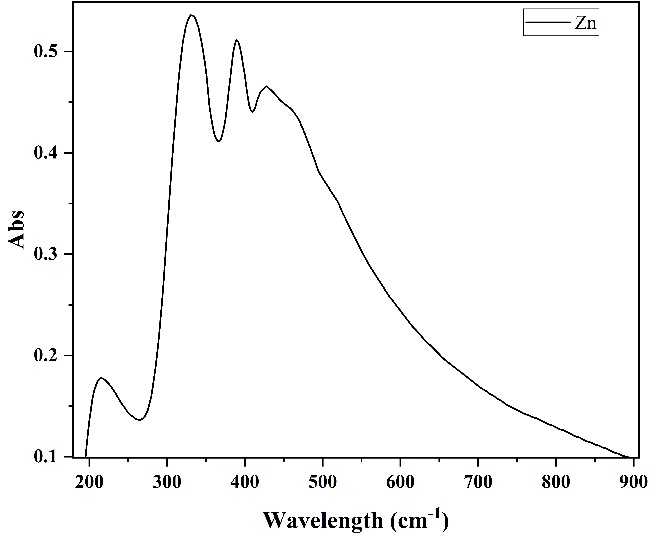** | |

**Figure S2**. Electronic spectra of H_2_L and its complexes.

| 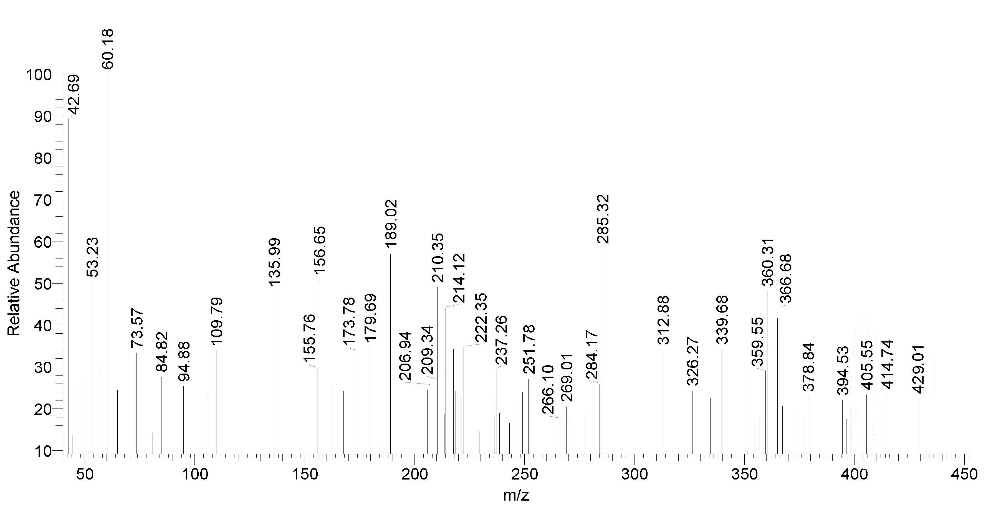 |
| --- |
| Co(II) |
| 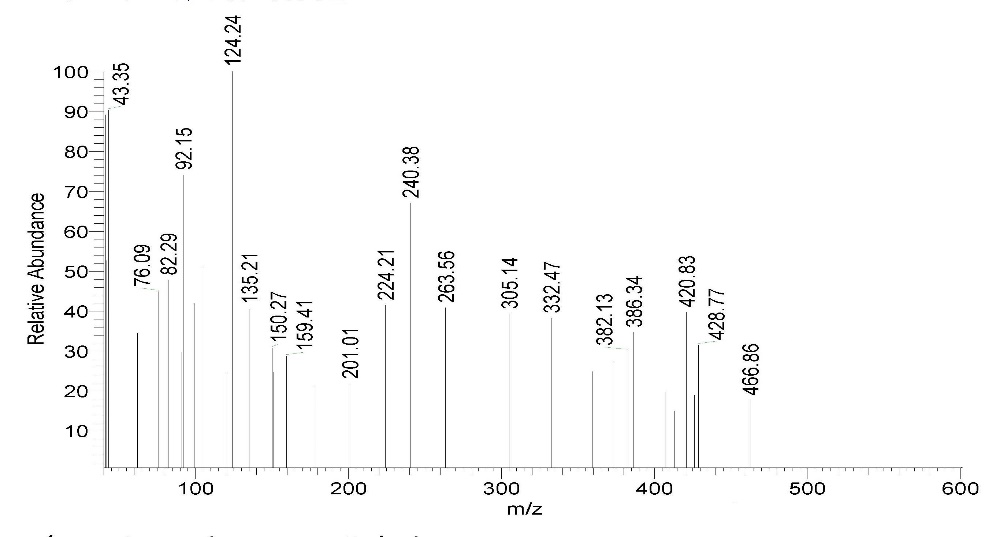 |
| Ni(II) |
| 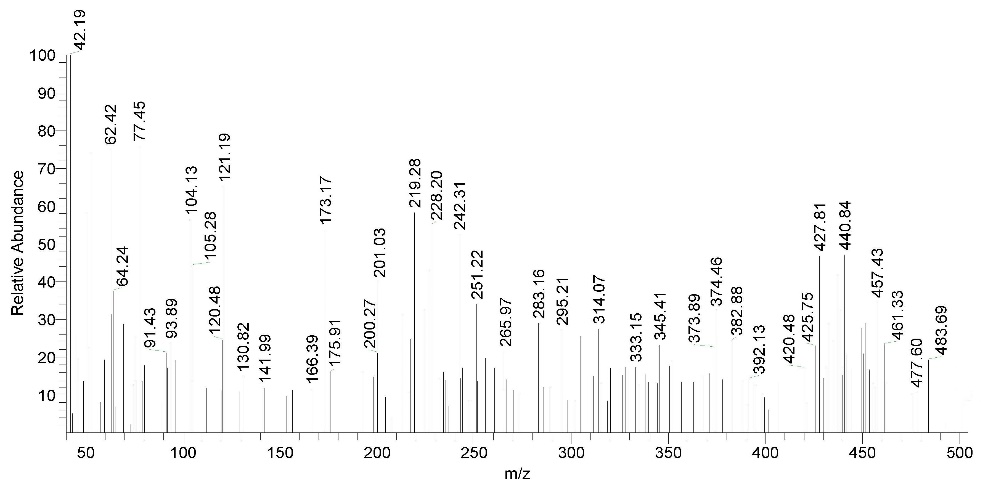 |
| Zn(II) |

**Figure S3**. Mass spectra of Co(II), Ni(II) and Zn(II) complexes.

| 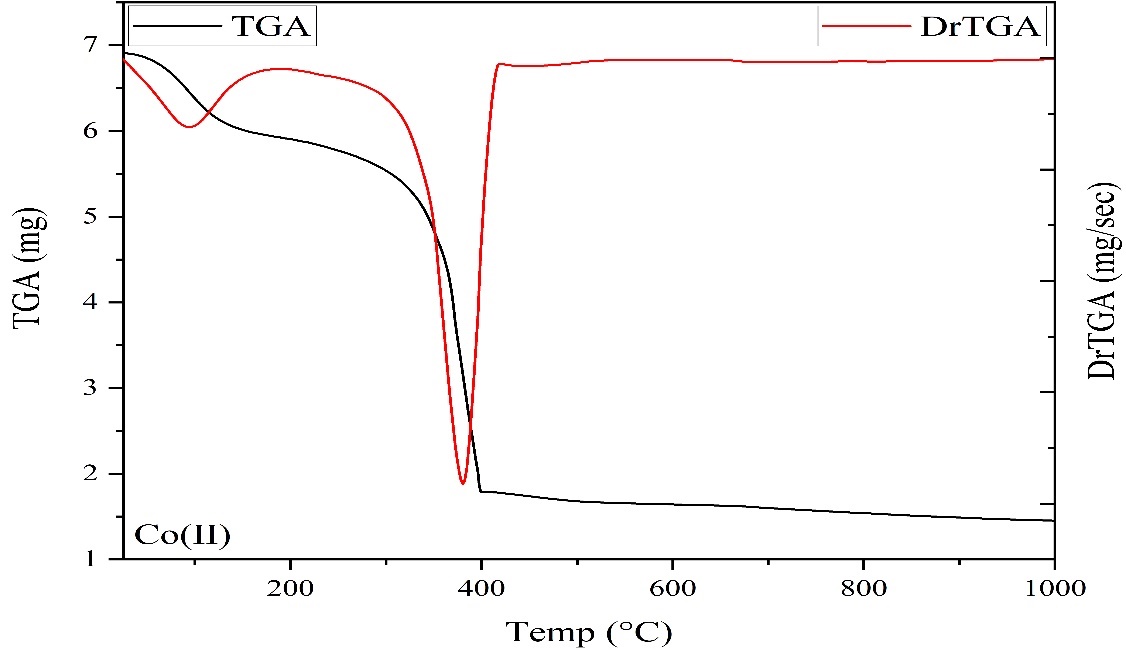 |
| --- |
| 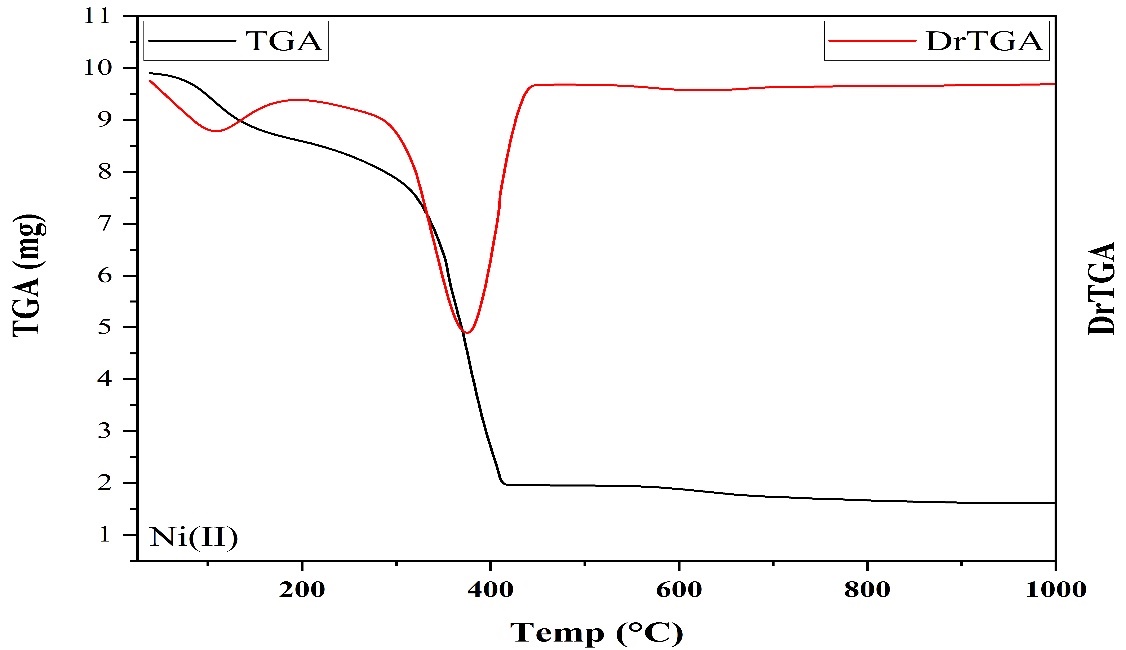 |
| 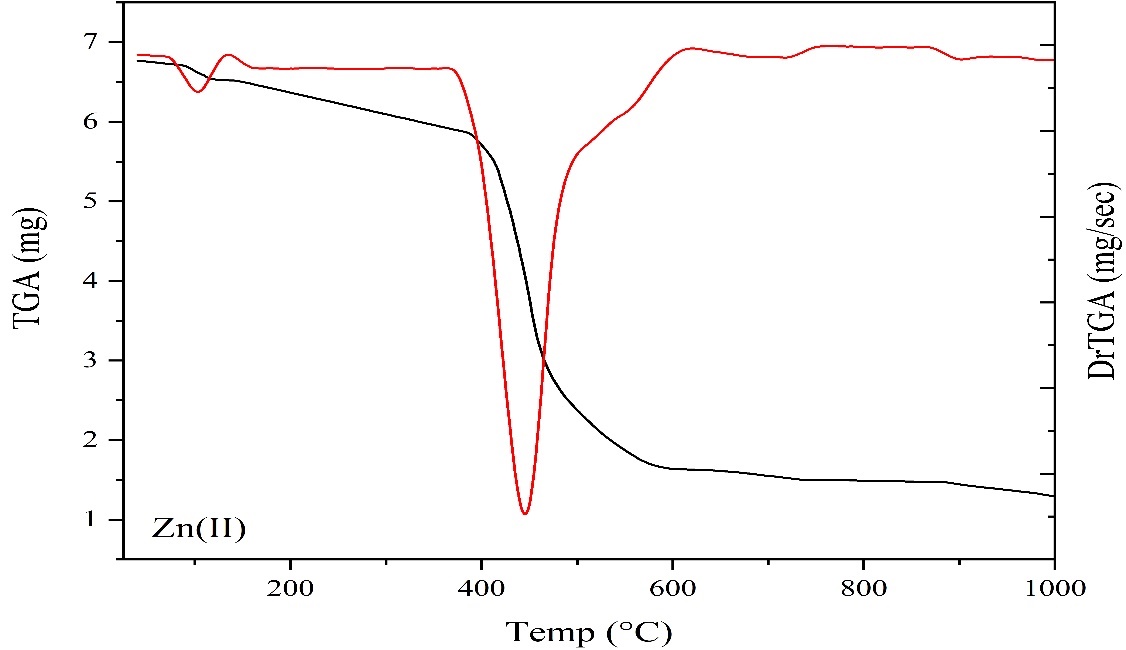 |

**Figure S4**. TGA curves of Co(II), Ni(II) and Zn(II) complexes.

**Table S1**. Selected DFT bond length of the ligand and its complexes.

| **H_2_L** | | **Cu(II) complex** | | **Co(II) complex** | | **Ni(II) complex** | | **Zn(II) complex** | |
| --- | --- | --- | --- | --- | --- | --- | --- | --- | --- |
| **Bond** | **Å** | **Bond** | **Å** | **Bond** | **Å** | **Bond** | **Å** | **Bond** | **Å** |
| C(14)-N(15) | 1.16 | C(19)-N(20) | 1.16 | C(22)-N(23) | 1.16 | C(26)-N(27) | 1.16 | C(16)-H(59) | 1.11 |
| C(13)-H(23) | 1.09 | C(17)-H(30) | 1.09 | O(21)-H(39) | 0.97 | C(25)-H(50) | 1.09 | O(15)-H(58) | 0.94 |
| C(13)-H(22) | 1.10 | C(17)-H(29) | 1.09 | O(21)-H(38) | 0.97 | C(25)-H(49) | 1.10 | C(28)-N(29) | 1.16 |
| C(13)-C(14) | 1.47 | C(17)-H(28) | 1.09 | O(20)-H(37) | 0.97 | C(25)-H(48) | 1.10 | C(27)-H(57) | 1.09 |
| C(11)-C(13) | 1.54 | C(16)-O(18) | 1.29 | O(20)-H(36) | 1.00 | C(24)-H(47) | 1.09 | C(27)-H(56) | 1.09 |
| C(11)-O(12) | 1.22 | C(16)-C(17) | 1.50 | O(19)-H(35) | 0.98 | C(24)-H(46) | 1.09 | C(27)-H(55) | 1.10 |
| N(10)-H(21) | 1.02 | O(15)-C(16) | 1.28 | O(19)-H(34) | 0.98 | C(24)-C(25) | 1.52 | C(26)-H(54) | 1.09 |
| N(10)-C(11) | 1.39 | Cu(14)-O(15) | 2.04 | C(17)-H(33) | 1.09 | C(23)-H(45) | 1.10 | C(26)-H(53) | 1.10 |
| N(9)-H(20) | 1.01 | Cu(14)-O(18) | 1.99 | C(17)-H(32) | 1.09 | C(23)-H(44) | 1.10 | C(26)-C(27) | 1.52 |
| N(9)-N(10) | 1.39 | C(13)-H(27) | 1.10 | C(17)-H(31) | 1.10 | C(23)-H(43) | 1.10 | C(25)-H(52) | 1.10 |
| C(7)-H(19) | 1.09 | C(13)-H(26) | 1.09 | C(16)-O(18) | 1.30 | C(22)-H(42) | 1.10 | C(25)-H(51) | 1.10 |
| C(6)-H(18) | 1.09 | C(13)-C(19) | 1.47 | C(16)-C(17) | 1.52 | C(22)-H(41) | 1.10 | C(25)-H(50) | 1.10 |
| C(6)-C(7) | 1.40 | O(12)-Cu(14) | 1.92 | O(15)-C(16) | 1.25 | C(22)-C(23) | 1.53 | C(24)-H(49) | 1.10 |
| N(5)-C(6) | 1.34 | C(11)-C(13) | 1.52 | Co(14)-O(20) | 2.03 | O(21)-H(51) | 1.03 | C(24)-H(48) | 1.09 |
| C(4)-H(17) | 1.09 | C(11)-O(12) | 1.30 | Co(14)-O(21) | 2.03 | O(21)-C(24) | 1.46 | C(24)-C(25) | 1.52 |
| C(4)-N(5) | 1.34 | N(10)-C(11) | 1.31 | Co(14)-O(19) | 2.28 | O(20)-H(40) | 1.00 | C(23)-H(47) | 1.09 |
| C(3)-H(16) | 1.09 | N(9)-Cu(14) | 2.08 | Co(14)-O(18) | 1.92 | O(20)-H(39) | 0.98 | C(23)-H(46) | 1.10 |
| C(3)-C(4) | 1.40 | N(9)-H(25) | 1.03 | C(13)-H(30) | 1.10 | O(19)-H(38) | 0.97 | C(23)-H(45) | 1.10 |
| C(2)-C(7) | 1.40 | N(9)-N(10) | 1.45 | C(13)-H(29) | 1.09 | O(19)-C(22) | 1.44 | C(22)-H(44) | 1.10 |
| C(2)-C(3) | 1.40 | C(7)-H(24) | 1.09 | C(13)-C(22) | 1.47 | C(17)-H(37) | 1.09 | C(22)-H(43) | 1.09 |
| C(1)-N(9) | 1.40 | C(6)-H(23) | 1.09 | O(12)-Co(14) | 1.92 | C(17)-H(36) | 1.09 | C(22)-C(23) | 1.52 |
| C(1)-O(8) | 1.22 | C(6)-C(7) | 1.40 | C(11)-C(13) | 1.53 | C(17)-H(35) | 1.09 | O(21)-H(42) | 0.98 |
| C(1)-C(2) | 1.50 | N(5)-C(6) | 1.34 | C(11)-O(12) | 1.30 | C(16)-O(18) | 1.29 | O(21)-C(24) | 1.45 |
|  |  | C(4)-H(22) | 1.09 | N(10)-C(11) | 1.30 | C(16)-C(17) | 1.52 | O(20)-H(41) | 1.02 |
|  |  | C(4)-N(5) | 1.34 | N(9)-Co(14) | 2.44 | O(15)-C(16) | 1.26 | O(20)-C(26) | 1.45 |
|  |  | C(3)-H(21) | 1.08 | N(9)-H(28) | 1.04 | Ni(14)-O(21) | 1.92 | O(19)-H(40) | 0.99 |
|  |  | C(3)-C(4) | 1.40 | N(9)-N(10) | 1.43 | Ni(14)-O(20) | 1.96 | O(19)-C(22) | 1.45 |
|  |  | C(2)-C(7) | 1.40 | C(7)-H(27) | 1.09 | Ni(14)-O(19) | 2.83 | C(17)-H(39) | 1.09 |
|  |  | C(2)-C(3) | 1.40 | C(6)-H(26) | 1.09 | Ni(14)-O(18) | 1.88 | C(17)-H(38) | 1.09 |
|  |  | C(1)-N(9) | 1.45 | C(6)-C(7) | 1.40 | C(13)-H(34) | 1.10 | C(17)-H(37) | 1.10 |
|  |  | C(1)-O(8) | 1.22 | N(5)-C(6) | 1.34 | C(13)-H(33) | 1.09 | C(16)-O(18) | 1.29 |
|  |  | C(1)-C(2) | 1.50 | C(4)-H(25) | 1.09 | C(13)-C(26) | 1.47 | C(16)-C(17) | 1.52 |
|  |  |  |  | C(4)-N(5) | 1.34 | O(12)-Ni(14) | 1.87 | O(15)-C(16) | 1.26 |
|  |  |  |  | C(3)-H(24) | 1.09 | C(11)-C(13) | 1.53 | Zn(14)-O(19) | 2.17 |
|  |  |  |  | C(3)-C(4) | 1.40 | C(11)-O(12) | 1.30 | Zn(14)-O(21) | 2.31 |
|  |  |  |  | C(2)-C(7) | 1.40 | N(10)-C(11) | 1.30 | Zn(14)-O(20) | 2.10 |
|  |  |  |  | C(2)-C(3) | 1.40 | N(9)-H(32) | 1.01 | Zn(14)-O(18) | 2.00 |
|  |  |  |  | C(1)-N(9) | 1.37 | N(9)-Ni(14) | 3.09 | C(13)-H(36) | 1.10 |
|  |  |  |  | C(1)-O(8) | 1.25 | N(9)-N(10) | 1.41 | C(13)-H(35) | 1.09 |
|  |  |  |  | C(1)-C(2) | 1.51 | C(7)-H(31) | 1.09 | C(13)-C(28) | 1.47 |
|  |  |  |  |  |  | C(6)-H(30) | 1.09 | O(12)-Zn(14) | 2.01 |
|  |  |  |  |  |  | C(6)-C(7) | 1.40 | C(11)-C(13) | 1.53 |
|  |  |  |  |  |  | N(5)-C(6) | 1.34 | C(11)-O(12) | 1.29 |
|  |  |  |  |  |  | C(4)-H(29) | 1.09 | N(10)-C(11) | 1.31 |
|  |  |  |  |  |  | C(4)-N(5) | 1.34 | N(9)-Zn(14) | 2.68 |
|  |  |  |  |  |  | C(3)-H(28) | 1.09 | N(9)-H(34) | 1.02 |
|  |  |  |  |  |  | C(3)-C(4) | 1.40 | N(9)-N(10) | 1.44 |
|  |  |  |  |  |  | C(2)-C(7) | 1.40 | C(7)-H(33) | 1.09 |
|  |  |  |  |  |  | C(2)-C(3) | 1.40 | C(6)-H(32) | 1.09 |
|  |  |  |  |  |  | C(1)-N(9) | 1.36 | C(6)-C(7) | 1.40 |
|  |  |  |  |  |  | C(1)-O(8) | 1.25 | N(5)-C(6) | 1.34 |
|  |  |  |  |  |  | C(1)-C(2) | 1.50 | C(4)-H(31) | 1.09 |
|  |  |  |  |  |  |  |  | C(4)-N(5) | 1.34 |
|  |  |  |  |  |  |  |  | C(3)-H(30) | 1.09 |
|  |  |  |  |  |  |  |  | C(3)-C(4) | 1.40 |
|  |  |  |  |  |  |  |  | C(2)-C(7) | 1.40 |
|  |  |  |  |  |  |  |  | C(2)-C(3) | 1.40 |
|  |  |  |  |  |  |  |  | C(1)-N(9) | 1.38 |
|  |  |  |  |  |  |  |  | C(1)-O(8) | 1.24 |
|  |  |  |  |  |  |  |  | C(1)-C(2) | 1.50 |

**Table S3**. Selected DFT bond angle of the complexes.

| **H_2_L** | | **Cu(II) complex** | | **Co(II) complex** | | **Ni(II) complex** | | **Zn(II) complex** | |
| --- | --- | --- | --- | --- | --- | --- | --- | --- | --- |
| **Angle** | **°** | **Angle** | **°** | **Angle** | **°** | **Angle** | **°** | **Angle** | **°** |
| N(15)-C(14)-C(13) | 178.6 | N(20)-C(19)-C(13) | 177.5 | N(23)-C(22)-C(13) | 177.9 | N(27)-C(26)-C(13) | 178.8 | N(29)-C(28)-C(13) | 179.3 |
| H(23)-C(13)-H(22) | 108.4 | C(16)-O(18)-Cu(14) | 89.7 | H(39)-O(21)-H(38) | 108.2 | H(50)-C(25)-H(49) | 108.5 | H(57)-C(27)-H(56) | 108.6 |
| H(23)-C(13)-C(14) | 109.0 | H(30)-C(17)-H(29) | 109.7 | H(39)-O(21)-Co(14) | 114.3 | H(50)-C(25)-H(48) | 108.4 | H(57)-C(27)-H(55) | 108.2 |
| H(23)-C(13)-C(11) | 106.1 | H(30)-C(17)-H(28) | 107.4 | H(38)-O(21)-Co(14) | 113.8 | H(50)-C(25)-C(24) | 111.1 | H(57)-C(27)-C(26) | 111.5 |
| H(22)-C(13)-C(14) | 108.4 | H(30)-C(17)-C(16) | 109.0 | H(37)-O(20)-H(36) | 107.7 | H(49)-C(25)-H(48) | 108.0 | H(56)-C(27)-H(55) | 108.3 |
| H(22)-C(13)-C(11) | 110.0 | H(29)-C(17)-H(28) | 110.4 | H(37)-O(20)-Co(14) | 112.6 | H(49)-C(25)-C(24) | 111.2 | H(56)-C(27)-C(26) | 111.0 |
| C(14)-C(13)-C(11) | 114.8 | H(29)-C(17)-C(16) | 110.7 | H(36)-O(20)-Co(14) | 110.8 | H(48)-C(25)-C(24) | 109.5 | H(55)-C(27)-C(26) | 109.2 |
| C(13)-C(11)-O(12) | 121.8 | H(28)-C(17)-C(16) | 109.5 | H(35)-O(19)-H(34) | 104.5 | H(47)-C(24)-H(46) | 107.8 | H(54)-C(26)-H(53) | 107.9 |
| C(13)-C(11)-N(10) | 117.0 | O(18)-C(16)-C(17) | 121.5 | H(35)-O(19)-Co(14) | 93.8 | H(47)-C(24)-C(25) | 112.0 | H(54)-C(26)-C(27) | 110.6 |
| O(12)-C(11)-N(10) | 121.0 | O(18)-C(16)-O(15) | 117.0 | H(34)-O(19)-Co(14) | 96.2 | H(47)-C(24)-O(21) | 108.9 | H(54)-C(26)-O(20) | 106.6 |
| H(21)-N(10)-C(11) | 113.1 | C(17)-C(16)-O(15) | 121.5 | C(16)-O(18)-Co(14) | 127.0 | H(46)-C(24)-C(25) | 112.0 | H(53)-C(26)-C(27) | 110.7 |
| H(21)-N(10)-N(9) | 115.6 | C(16)-O(15)-Cu(14) | 87.7 | H(33)-C(17)-H(32) | 110.6 | H(46)-C(24)-O(21) | 106.1 | H(53)-C(26)-O(20) | 107.3 |
| C(11)-N(10)-N(9) | 121.4 | O(15)-Cu(14)-O(18) | 65.6 | H(33)-C(17)-H(31) | 107.7 | C(25)-C(24)-O(21) | 109.9 | C(27)-C(26)-O(20) | 113.5 |
| H(20)-N(9)-N(10) | 115.4 | O(15)-Cu(14)-O(12) | 107.3 | H(33)-C(17)-C(16) | 111.1 | H(45)-C(23)-H(44) | 108.5 | H(52)-C(25)-H(51) | 108.4 |
| H(20)-N(9)-C(1) | 120.1 | O(15)-Cu(14)-N(9) | 171.0 | H(32)-C(17)-H(31) | 108.5 | H(45)-C(23)-H(43) | 108.4 | H(52)-C(25)-H(50) | 108.3 |
| N(10)-N(9)-C(1) | 119.4 | O(18)-Cu(14)-O(12) | 172.7 | H(32)-C(17)-C(16) | 110.3 | H(45)-C(23)-C(22) | 110.6 | H(52)-C(25)-C(24) | 111.0 |
| H(19)-C(7)-C(6) | 121.3 | O(18)-Cu(14)-N(9) | 105.6 | H(31)-C(17)-C(16) | 108.6 | H(44)-C(23)-H(43) | 107.8 | H(51)-C(25)-H(50) | 107.7 |
| H(19)-C(7)-C(2) | 120.1 | O(12)-Cu(14)-N(9) | 81.5 | O(18)-C(16)-C(17) | 114.4 | H(44)-C(23)-C(22) | 111.1 | H(51)-C(25)-C(24) | 111.4 |
| C(6)-C(7)-C(2) | 118.6 | H(27)-C(13)-H(26) | 106.6 | O(18)-C(16)-O(15) | 125.5 | H(43)-C(23)-C(22) | 110.3 | H(50)-C(25)-C(24) | 109.9 |
| H(18)-C(6)-C(7) | 120.1 | H(27)-C(13)-C(19) | 109.4 | C(17)-C(16)-O(15) | 120.1 | H(42)-C(22)-H(41) | 107.7 | H(49)-C(24)-H(48) | 107.6 |
| H(18)-C(6)-N(5) | 116.1 | H(27)-C(13)-C(11) | 108.3 | O(20)-Co(14)-O(21) | 86.3 | H(42)-C(22)-C(23) | 110.6 | H(49)-C(24)-C(25) | 111.5 |
| C(7)-C(6)-N(5) | 123.8 | H(26)-C(13)-C(19) | 109.3 | O(20)-Co(14)-O(19) | 94.0 | H(42)-C(22)-O(19) | 110.0 | H(49)-C(24)-O(21) | 109.4 |
| C(6)-N(5)-C(4) | 117.2 | H(26)-C(13)-C(11) | 108.0 | O(20)-Co(14)-O(18) | 172.4 | H(41)-C(22)-C(23) | 110.3 | H(48)-C(24)-C(25) | 111.5 |
| H(17)-C(4)-N(5) | 116.2 | C(19)-C(13)-C(11) | 115.0 | O(20)-Co(14)-O(12) | 91.6 | H(41)-C(22)-O(19) | 105.3 | H(48)-C(24)-O(21) | 105.0 |
| H(17)-C(4)-C(3) | 120.1 | Cu(14)-O(12)-C(11) | 111.5 | O(20)-Co(14)-N(9) | 85.3 | C(23)-C(22)-O(19) | 112.7 | C(25)-C(24)-O(21) | 111.6 |
| N(5)-C(4)-C(3) | 123.7 | C(13)-C(11)-O(12) | 114.6 | O(21)-Co(14)-O(19) | 93.1 | H(51)-O(21)-C(24) | 108.7 | H(47)-C(23)-H(46) | 109.0 |
| H(16)-C(3)-C(4) | 119.0 | C(13)-C(11)-N(10) | 117.8 | O(21)-Co(14)-O(18) | 87.1 | H(51)-O(21)-Ni(14) | 99.6 | H(47)-C(23)-H(45) | 108.4 |
| H(16)-C(3)-C(2) | 122.4 | O(12)-C(11)-N(10) | 127.6 | O(21)-Co(14)-O(12) | 176.5 | C(24)-O(21)-Ni(14) | 116.1 | H(47)-C(23)-C(22) | 110.6 |
| C(4)-C(3)-C(2) | 118.6 | C(11)-N(10)-N(9) | 109.9 | O(21)-Co(14)-N(9) | 107.4 | H(40)-O(20)-H(39) | 106.1 | H(46)-C(23)-H(45) | 107.9 |
| C(7)-C(2)-C(3) | 118.1 | Cu(14)-N(9)-H(25) | 104.1 | O(19)-Co(14)-O(18) | 82.5 | H(40)-O(20)-Ni(14) | 104.5 | H(46)-C(23)-C(22) | 111.2 |
| C(7)-C(2)-C(1) | 117.9 | Cu(14)-N(9)-N(10) | 108.7 | O(19)-Co(14)-O(12) | 84.2 | H(39)-O(20)-Ni(14) | 108.5 | H(45)-C(23)-C(22) | 109.7 |
| C(3)-C(2)-C(1) | 124.0 | Cu(14)-N(9)-C(1) | 111.9 | O(19)-Co(14)-N(9) | 159.4 | H(38)-O(19)-C(22) | 107.6 | H(44)-C(22)-H(43) | 108.5 |
| N(9)-C(1)-O(8) | 121.4 | H(25)-N(9)-N(10) | 108.5 | O(18)-Co(14)-O(12) | 94.8 | H(38)-O(19)-Ni(14) | 70.0 | H(44)-C(22)-C(23) | 111.2 |
| N(9)-C(1)-C(2) | 115.4 | H(25)-N(9)-C(1) | 104.7 | O(18)-Co(14)-N(9) | 100.3 | C(22)-O(19)-Ni(14) | 124.1 | H(44)-C(22)-O(19) | 108.0 |
| O(8)-C(1)-C(2) | 123.2 | N(10)-N(9)-C(1) | 117.9 | O(12)-Co(14)-N(9) | 75.2 | C(16)-O(18)-Ni(14) | 124.5 | H(43)-C(22)-C(23) | 110.9 |
|  |  | H(24)-C(7)-C(6) | 121.0 | H(30)-C(13)-H(29) | 108.9 | H(37)-C(17)-H(36) | 109.5 | H(43)-C(22)-O(19) | 105.1 |
|  |  | H(24)-C(7)-C(2) | 120.4 | H(30)-C(13)-C(22) | 108.8 | H(37)-C(17)-H(35) | 107.3 | C(23)-C(22)-O(19) | 112.9 |
|  |  | C(6)-C(7)-C(2) | 118.7 | H(30)-C(13)-C(11) | 109.3 | H(37)-C(17)-C(16) | 109.1 | H(42)-O(21)-C(24) | 109.4 |
|  |  | H(23)-C(6)-C(7) | 120.2 | H(29)-C(13)-C(22) | 109.8 | H(36)-C(17)-H(35) | 110.5 | H(42)-O(21)-Zn(14) | 94.8 |
|  |  | H(23)-C(6)-N(5) | 116.2 | H(29)-C(13)-C(11) | 110.0 | H(36)-C(17)-C(16) | 110.3 | C(24)-O(21)-Zn(14) | 129.3 |
|  |  | C(7)-C(6)-N(5) | 123.6 | C(22)-C(13)-C(11) | 110.0 | H(35)-C(17)-C(16) | 110.2 | H(41)-O(20)-C(26) | 109.7 |
|  |  | C(6)-N(5)-C(4) | 117.2 | Co(14)-O(12)-C(11) | 115.8 | O(18)-C(16)-C(17) | 114.8 | H(41)-O(20)-Zn(14) | 94.9 |
|  |  | H(22)-C(4)-N(5) | 116.2 | C(13)-C(11)-O(12) | 115.7 | O(18)-C(16)-O(15) | 125.1 | C(26)-O(20)-Zn(14) | 128.9 |
|  |  | H(22)-C(4)-C(3) | 119.7 | C(13)-C(11)-N(10) | 115.2 | C(17)-C(16)-O(15) | 120.1 | H(40)-O(19)-C(22) | 111.1 |
|  |  | N(5)-C(4)-C(3) | 124.1 | O(12)-C(11)-N(10) | 129.0 | O(21)-Ni(14)-O(20) | 87.9 | H(40)-O(19)-Zn(14) | 115.0 |
|  |  | H(21)-C(3)-C(4) | 120.4 | C(11)-N(10)-N(9) | 112.5 | O(21)-Ni(14)-O(19) | 102.3 | C(22)-O(19)-Zn(14) | 124.7 |
|  |  | H(21)-C(3)-C(2) | 121.5 | Co(14)-N(9)-H(28) | 90.7 | O(21)-Ni(14)-O(18) | 95.1 | C(16)-O(18)-Zn(14) | 123.4 |
|  |  | C(4)-C(3)-C(2) | 118.1 | Co(14)-N(9)-N(10) | 101.3 | O(21)-Ni(14)-O(12) | 174.1 | H(39)-C(17)-H(38) | 110.5 |
|  |  | C(7)-C(2)-C(3) | 118.3 | Co(14)-N(9)-C(1) | 102.9 | O(21)-Ni(14)-N(9) | 119.8 | H(39)-C(17)-H(37) | 109.2 |
|  |  | C(7)-C(2)-C(1) | 116.5 | H(28)-N(9)-N(10) | 114.1 | O(20)-Ni(14)-O(19) | 86.0 | H(39)-C(17)-C(16) | 110.8 |
|  |  | C(3)-C(2)-C(1) | 125.2 | H(28)-N(9)-C(1) | 121.5 | O(20)-Ni(14)-O(18) | 175.0 | H(38)-C(17)-H(37) | 107.1 |
|  |  | N(9)-C(1)-O(8) | 117.1 | N(10)-N(9)-C(1) | 118.2 | O(20)-Ni(14)-O(12) | 87.3 | H(38)-C(17)-C(16) | 110.1 |
|  |  | N(9)-C(1)-C(2) | 119.6 | H(27)-C(7)-C(6) | 121.2 | O(20)-Ni(14)-N(9) | 93.9 | H(37)-C(17)-C(16) | 109.1 |
|  |  | O(8)-C(1)-C(2) | 122.9 | H(27)-C(7)-C(2) | 119.7 | O(19)-Ni(14)-O(18) | 97.1 | H(59)-C(16)-O(18) | 86.4 |
|  |  |  |  | C(6)-C(7)-C(2) | 119.0 | O(19)-Ni(14)-O(12) | 73.8 | H(59)-C(16)-C(17) | 157.3 |
|  |  |  |  | H(26)-C(6)-C(7) | 120.2 | O(19)-Ni(14)-N(9) | 137.9 | H(59)-C(16)-O(15) | 39.0 |
|  |  |  |  | H(26)-C(6)-N(5) | 116.1 | O(18)-Ni(14)-O(12) | 89.8 | O(18)-C(16)-C(17) | 115.6 |
|  |  |  |  | C(7)-C(6)-N(5) | 123.7 | O(18)-Ni(14)-N(9) | 81.2 | O(18)-C(16)-O(15) | 125.0 |
|  |  |  |  | C(6)-N(5)-C(4) | 116.9 | O(12)-Ni(14)-N(9) | 64.1 | C(17)-C(16)-O(15) | 119.4 |
|  |  |  |  | H(25)-C(4)-N(5) | 116.1 | H(34)-C(13)-H(33) | 108.1 | H(58)-O(15)-C(16) | 106.9 |
|  |  |  |  | H(25)-C(4)-C(3) | 119.8 | H(34)-C(13)-C(26) | 107.9 | O(19)-Zn(14)-O(21) | 159.0 |
|  |  |  |  | N(5)-C(4)-C(3) | 124.1 | H(34)-C(13)-C(11) | 108.0 | O(19)-Zn(14)-O(20) | 94.0 |
|  |  |  |  | H(24)-C(3)-C(4) | 119.0 | H(33)-C(13)-C(26) | 109.9 | O(19)-Zn(14)-O(18) | 100.3 |
|  |  |  |  | H(24)-C(3)-C(2) | 122.6 | H(33)-C(13)-C(11) | 109.8 | O(19)-Zn(14)-O(12) | 96.1 |
|  |  |  |  | C(4)-C(3)-C(2) | 118.5 | C(26)-C(13)-C(11) | 113.0 | O(19)-Zn(14)-N(9) | 80.1 |
|  |  |  |  | C(7)-C(2)-C(3) | 117.7 | Ni(14)-O(12)-C(11) | 129.8 | O(21)-Zn(14)-O(20) | 85.4 |
|  |  |  |  | C(7)-C(2)-C(1) | 117.4 | C(13)-C(11)-O(12) | 114.5 | O(21)-Zn(14)-O(18) | 100.7 |
|  |  |  |  | C(3)-C(2)-C(1) | 124.8 | C(13)-C(11)-N(10) | 113.1 | O(21)-Zn(14)-O(12) | 77.7 |
|  |  |  |  | N(9)-C(1)-O(8) | 121.4 | O(12)-C(11)-N(10) | 132.3 | O(21)-Zn(14)-N(9) | 78.9 |
|  |  |  |  | N(9)-C(1)-C(2) | 118.1 | C(11)-N(10)-N(9) | 116.9 | O(20)-Zn(14)-O(18) | 96.4 |
|  |  |  |  | O(8)-C(1)-C(2) | 120.3 | H(32)-N(9)-Ni(14) | 123.9 | O(20)-Zn(14)-O(12) | 156.6 |
|  |  |  |  |  |  | H(32)-N(9)-N(10) | 111.7 | O(20)-Zn(14)-N(9) | 92.3 |
|  |  |  |  |  |  | H(32)-N(9)-C(1) | 116.5 | O(18)-Zn(14)-O(12) | 102.6 |
|  |  |  |  |  |  | Ni(14)-N(9)-N(10) | 92.0 | O(18)-Zn(14)-N(9) | 171.2 |
|  |  |  |  |  |  | Ni(14)-N(9)-C(1) | 83.3 | O(12)-Zn(14)-N(9) | 68.7 |
|  |  |  |  |  |  | N(10)-N(9)-C(1) | 124.5 | H(36)-C(13)-H(35) | 109.1 |
|  |  |  |  |  |  | H(31)-C(7)-C(6) | 121.1 | H(36)-C(13)-C(28) | 108.3 |
|  |  |  |  |  |  | H(31)-C(7)-C(2) | 120.2 | H(36)-C(13)-C(11) | 109.0 |
|  |  |  |  |  |  | C(6)-C(7)-C(2) | 118.7 | H(35)-C(13)-C(28) | 109.3 |
|  |  |  |  |  |  | H(30)-C(6)-C(7) | 120.1 | H(35)-C(13)-C(11) | 109.9 |
|  |  |  |  |  |  | H(30)-C(6)-N(5) | 116.1 | C(28)-C(13)-C(11) | 111.1 |
|  |  |  |  |  |  | C(7)-C(6)-N(5) | 123.8 | Zn(14)-O(12)-C(11) | 121.3 |
|  |  |  |  |  |  | C(6)-N(5)-C(4) | 117.1 | C(13)-C(11)-O(12) | 116.3 |
|  |  |  |  |  |  | H(29)-C(4)-N(5) | 116.2 | C(13)-C(11)-N(10) | 113.9 |
|  |  |  |  |  |  | H(29)-C(4)-C(3) | 119.9 | O(12)-C(11)-N(10) | 129.8 |
|  |  |  |  |  |  | N(5)-C(4)-C(3) | 123.9 | C(11)-N(10)-N(9) | 112.4 |
|  |  |  |  |  |  | H(28)-C(3)-C(4) | 119.5 | Zn(14)-N(9)-H(34) | 101.4 |
|  |  |  |  |  |  | H(28)-C(3)-C(2) | 121.9 | Zn(14)-N(9)-N(10) | 101.0 |
|  |  |  |  |  |  | C(4)-C(3)-C(2) | 118.5 | Zn(14)-N(9)-C(1) | 108.3 |
|  |  |  |  |  |  | C(7)-C(2)-C(3) | 118.1 | H(34)-N(9)-N(10) | 111.4 |
|  |  |  |  |  |  | C(7)-C(2)-C(1) | 118.7 | H(34)-N(9)-C(1) | 115.3 |
|  |  |  |  |  |  | C(3)-C(2)-C(1) | 123.2 | N(10)-N(9)-C(1) | 117.1 |
|  |  |  |  |  |  | N(9)-C(1)-O(8) | 123.6 | H(33)-C(7)-C(6) | 119.1 |
|  |  |  |  |  |  | N(9)-C(1)-C(2) | 115.3 | H(33)-C(7)-C(2) | 122.2 |
|  |  |  |  |  |  | O(8)-C(1)-C(2) | 121.2 | C(6)-C(7)-C(2) | 118.6 |
|  |  |  |  |  |  |  |  | H(32)-C(6)-C(7) | 120.0 |
|  |  |  |  |  |  |  |  | H(32)-C(6)-N(5) | 116.1 |
|  |  |  |  |  |  |  |  | C(7)-C(6)-N(5) | 123.9 |
|  |  |  |  |  |  |  |  | C(6)-N(5)-C(4) | 117.0 |
|  |  |  |  |  |  |  |  | H(31)-C(4)-N(5) | 116.1 |
|  |  |  |  |  |  |  |  | H(31)-C(4)-C(3) | 120.1 |
|  |  |  |  |  |  |  |  | N(5)-C(4)-C(3) | 123.7 |
|  |  |  |  |  |  |  |  | H(30)-C(3)-C(4) | 121.2 |
|  |  |  |  |  |  |  |  | H(30)-C(3)-C(2) | 120.0 |
|  |  |  |  |  |  |  |  | C(4)-C(3)-C(2) | 118.8 |
|  |  |  |  |  |  |  |  | C(7)-C(2)-C(3) | 117.9 |
|  |  |  |  |  |  |  |  | C(7)-C(2)-C(1) | 124.3 |
|  |  |  |  |  |  |  |  | C(3)-C(2)-C(1) | 117.8 |
|  |  |  |  |  |  |  |  | N(9)-C(1)-O(8) | 121.6 |
|  |  |  |  |  |  |  |  | N(9)-C(1)-C(2) | 117.1 |
|  |  |  |  |  |  |  |  | O(8)-C(1)-C(2) | 121.3 |

**Table S4**. Selected DFT dihedral angle of the ligand and its complexes.

| **H_2_L** | | **Cu(II) complex** | | **Co(II) complex** | | **Ni(II) complex** | | **Zn(II) complex** | |
| --- | --- | --- | --- | --- | --- | --- | --- | --- | --- |
| **Dihedral Angle** | **°** | **Dihedral Angle** | **°** | **Dihedral Angle** | **°** | **Dihedral Angle** | **°** | **Dihedral Angle** | **°** |
| C(11)-C(13)-C(14)-N(15) | 162.7 | O(15)-C(16)-O(18)-Cu(14) | -0.2 | O(15)-C(16)-O(18)-Co(14) | 11.1 | O(21)-C(24)-C(25)-H(48) | 178.8 | O(20)-C(26)-C(27)-H(55) | 179.5 |
| H(22)-C(13)-C(14)-N(15) | -73.9 | C(17)-C(16)-O(18)-Cu(14) | -179.8 | C(17)-C(16)-O(18)-Co(14) | -168.5 | O(21)-C(24)-C(25)-H(49) | -61.9 | O(20)-C(26)-C(27)-H(56) | -61.2 |
| H(23)-C(13)-C(14)-N(15) | 43.9 | O(15)-C(16)-C(17)-H(28) | 52.4 | O(15)-C(16)-C(17)-H(31) | -97.9 | O(21)-C(24)-C(25)-H(50) | 59.1 | O(20)-C(26)-C(27)-H(57) | 60.1 |
| N(10)-C(11)-C(13)-C(14) | 72.0 | O(15)-C(16)-C(17)-H(29) | 174.4 | O(15)-C(16)-C(17)-H(32) | 20.8 | H(46)-C(24)-C(25)-H(48) | 61.2 | H(53)-C(26)-C(27)-H(55) | 58.8 |
| N(10)-C(11)-C(13)-H(22) | -50.5 | O(15)-C(16)-C(17)-H(30) | -64.9 | O(15)-C(16)-C(17)-H(33) | 143.8 | H(46)-C(24)-C(25)-H(49) | -179.5 | H(53)-C(26)-C(27)-H(56) | 178.1 |
| N(10)-C(11)-C(13)-H(23) | -167.5 | O(18)-C(16)-C(17)-H(28) | -128.0 | O(18)-C(16)-C(17)-H(31) | 81.7 | H(46)-C(24)-C(25)-H(50) | -58.5 | H(53)-C(26)-C(27)-H(57) | -60.7 |
| O(12)-C(11)-C(13)-C(14) | -114.1 | O(18)-C(16)-C(17)-H(29) | -6.0 | O(18)-C(16)-C(17)-H(32) | -159.5 | H(47)-C(24)-C(25)-H(48) | -60.1 | H(54)-C(26)-C(27)-H(55) | -60.8 |
| O(12)-C(11)-C(13)-H(22) | 123.4 | O(18)-C(16)-C(17)-H(30) | 114.7 | O(18)-C(16)-C(17)-H(33) | -36.5 | H(47)-C(24)-C(25)-H(49) | 59.2 | H(54)-C(26)-C(27)-H(56) | 58.5 |
| O(12)-C(11)-C(13)-H(23) | 6.4 | Cu(14)-O(15)-C(16)-C(17) | 179.8 | N(9)-Co(14)-O(20)-H(36) | -7.0 | H(47)-C(24)-C(25)-H(50) | -179.8 | H(54)-C(26)-C(27)-H(57) | 179.8 |
| N(9)-N(10)-C(11)-O(12) | 163.9 | Cu(14)-O(15)-C(16)-O(18) | 0.2 | N(9)-Co(14)-O(20)-H(37) | 113.7 | O(19)-C(22)-C(23)-H(43) | 176.9 | O(21)-C(24)-C(25)-H(50) | 178.0 |
| N(9)-N(10)-C(11)-C(13) | -22.1 | N(9)-Cu(14)-O(15)-C(16) | -15.9 | O(12)-Co(14)-O(20)-H(36) | -82.0 | O(19)-C(22)-C(23)-H(44) | -63.6 | O(21)-C(24)-C(25)-H(51) | -62.8 |
| H(21)-N(10)-C(11)-O(12) | 19.7 | O(12)-Cu(14)-O(15)-C(16) | 177.9 | O(12)-Co(14)-O(20)-H(37) | 38.7 | O(19)-C(22)-C(23)-H(45) | 57.0 | O(21)-C(24)-C(25)-H(52) | 58.1 |
| H(21)-N(10)-C(11)-C(13) | -166.4 | O(18)-Cu(14)-O(15)-C(16) | -0.1 | O(18)-Co(14)-O(20)-H(36) | 131.0 | H(41)-C(22)-C(23)-H(43) | 59.6 | H(48)-C(24)-C(25)-H(50) | 60.9 |
| C(1)-N(9)-N(10)-C(11) | -94.4 | N(9)-Cu(14)-O(18)-C(16) | 177.6 | O(18)-Co(14)-O(20)-H(37) | -108.3 | H(41)-C(22)-C(23)-H(44) | 179.1 | H(48)-C(24)-C(25)-H(51) | -179.9 |
| C(1)-N(9)-N(10)-H(21) | 49.0 | O(12)-Cu(14)-O(18)-C(16) | -15.5 | O(19)-Co(14)-O(20)-H(36) | -166.3 | H(41)-C(22)-C(23)-H(45) | -60.3 | H(48)-C(24)-C(25)-H(52) | -59.0 |
| H(20)-N(9)-N(10)-C(11) | 110.9 | O(15)-Cu(14)-O(18)-C(16) | 0.1 | O(19)-Co(14)-O(20)-H(37) | -45.6 | H(42)-C(22)-C(23)-H(43) | -59.5 | H(49)-C(24)-C(25)-H(50) | -59.5 |
| H(20)-N(9)-N(10)-H(21) | -105.7 | C(11)-C(13)-C(19)-N(20) | 147.2 | O(21)-Co(14)-O(20)-H(36) | 100.8 | H(42)-C(22)-C(23)-H(44) | 60.0 | H(49)-C(24)-C(25)-H(51) | 59.8 |
| N(5)-C(6)-C(7)-C(2) | 0.9 | H(26)-C(13)-C(19)-N(20) | 25.6 | O(21)-Co(14)-O(20)-H(37) | -138.5 | H(42)-C(22)-C(23)-H(45) | -179.4 | H(49)-C(24)-C(25)-H(52) | -179.3 |
| N(5)-C(6)-C(7)-H(19) | -179.5 | H(27)-C(13)-C(19)-N(20) | -90.7 | N(9)-Co(14)-O(21)-H(38) | -59.2 | Ni(14)-O(21)-C(24)-C(25) | -178.6 | O(19)-C(22)-C(23)-H(45) | -179.4 |
| H(18)-C(6)-C(7)-C(2) | -179.2 | C(11)-O(12)-Cu(14)-N(9) | 7.1 | N(9)-Co(14)-O(21)-H(39) | 65.8 | Ni(14)-O(21)-C(24)-H(46) | -57.3 | O(19)-C(22)-C(23)-H(46) | -60.1 |
| H(18)-C(6)-C(7)-H(19) | 0.4 | C(11)-O(12)-Cu(14)-O(18) | -160.3 | O(12)-Co(14)-O(21)-H(38) | 162.7 | Ni(14)-O(21)-C(24)-H(47) | 58.4 | O(19)-C(22)-C(23)-H(47) | 61.1 |
| C(4)-N(5)-C(6)-C(7) | -0.1 | C(11)-O(12)-Cu(14)-O(15) | -175.1 | O(12)-Co(14)-O(21)-H(39) | -72.3 | H(51)-O(21)-C(24)-C(25) | 70.2 | H(43)-C(22)-C(23)-H(45) | 63.0 |
| C(4)-N(5)-C(6)-H(18) | -180.0 | N(10)-C(11)-C(13)-C(19) | 14.0 | O(18)-Co(14)-O(21)-H(38) | 40.7 | H(51)-O(21)-C(24)-H(46) | -168.5 | H(43)-C(22)-C(23)-H(46) | -177.7 |
| C(3)-C(4)-N(5)-C(6) | -0.7 | N(10)-C(11)-C(13)-H(26) | 136.3 | O(18)-Co(14)-O(21)-H(39) | 165.7 | H(51)-O(21)-C(24)-H(47) | -52.8 | H(43)-C(22)-C(23)-H(47) | -56.5 |
| H(17)-C(4)-N(5)-C(6) | 179.1 | N(10)-C(11)-C(13)-H(27) | -108.7 | O(19)-Co(14)-O(21)-H(38) | 123.0 | Ni(14)-O(19)-C(22)-C(23) | 140.0 | H(44)-C(22)-C(23)-H(45) | -57.8 |
| C(2)-C(3)-C(4)-N(5) | 0.6 | O(12)-C(11)-C(13)-C(19) | -166.6 | O(19)-Co(14)-O(21)-H(39) | -112.0 | Ni(14)-O(19)-C(22)-H(41) | -99.7 | H(44)-C(22)-C(23)-H(46) | 61.5 |
| C(2)-C(3)-C(4)-H(17) | -179.1 | O(12)-C(11)-C(13)-H(26) | -44.3 | O(20)-Co(14)-O(21)-H(38) | -143.2 | Ni(14)-O(19)-C(22)-H(42) | 16.1 | H(44)-C(22)-C(23)-H(47) | -177.3 |
| H(16)-C(3)-C(4)-N(5) | 178.2 | O(12)-C(11)-C(13)-H(27) | 70.7 | O(20)-Co(14)-O(21)-H(39) | -18.2 | H(38)-O(19)-C(22)-C(23) | 62.6 | Zn(14)-O(21)-C(24)-C(25) | 177.0 |
| H(16)-C(3)-C(4)-H(17) | -1.6 | N(10)-C(11)-O(12)-Cu(14) | -4.9 | N(9)-Co(14)-O(19)-H(34) | 5.0 | H(38)-O(19)-C(22)-H(41) | -177.1 | Zn(14)-O(21)-C(24)-H(48) | -62.1 |
| C(1)-C(2)-C(7)-C(6) | -179.4 | C(13)-C(11)-O(12)-Cu(14) | 175.8 | N(9)-Co(14)-O(19)-H(35) | 110.1 | H(38)-O(19)-C(22)-H(42) | -61.3 | Zn(14)-O(21)-C(24)-H(49) | 53.2 |
| C(1)-C(2)-C(7)-H(19) | 1.0 | N(9)-N(10)-C(11)-O(12) | -2.5 | O(12)-Co(14)-O(19)-H(34) | 1.3 | O(15)-C(16)-O(18)-Ni(14) | 5.5 | H(42)-O(21)-C(24)-C(25) | 63.3 |
| C(3)-C(2)-C(7)-C(6) | -0.9 | N(9)-N(10)-C(11)-C(13) | 176.8 | O(12)-Co(14)-O(19)-H(35) | 106.4 | C(17)-C(16)-O(18)-Ni(14) | -175.1 | H(42)-O(21)-C(24)-H(48) | -175.7 |
| C(3)-C(2)-C(7)-H(19) | 179.4 | C(1)-N(9)-Cu(14)-O(12) | -140.2 | O(18)-Co(14)-O(19)-H(34) | -94.3 | O(15)-C(16)-C(17)-H(35) | -129.4 | H(42)-O(21)-C(24)-H(49) | -60.5 |
| C(1)-C(2)-C(3)-C(4) | 178.6 | C(1)-N(9)-Cu(14)-O(18) | 38.1 | O(18)-Co(14)-O(19)-H(35) | 10.8 | O(15)-C(16)-C(17)-H(36) | -7.2 | Zn(14)-O(20)-C(26)-C(27) | 75.2 |
| C(1)-C(2)-C(3)-H(16) | 1.1 | C(1)-N(9)-Cu(14)-O(15) | 53.0 | O(21)-Co(14)-O(19)-H(34) | 179.1 | O(15)-C(16)-C(17)-H(37) | 113.1 | Zn(14)-O(20)-C(26)-H(53) | -162.1 |
| C(7)-C(2)-C(3)-C(4) | 0.3 | N(10)-N(9)-Cu(14)-O(12) | -8.2 | O(21)-Co(14)-O(19)-H(35) | -75.8 | O(18)-C(16)-C(17)-H(35) | 51.2 | Zn(14)-O(20)-C(26)-H(54) | -46.7 |
| C(7)-C(2)-C(3)-H(16) | -177.2 | N(10)-N(9)-Cu(14)-O(18) | 170.1 | O(20)-Co(14)-O(19)-H(34) | 92.5 | O(18)-C(16)-C(17)-H(36) | 173.4 | H(41)-O(20)-C(26)-C(27) | -38.7 |
| C(2)-C(1)-N(9)-N(10) | -170.9 | N(10)-N(9)-Cu(14)-O(15) | -175.0 | O(20)-Co(14)-O(19)-H(35) | -162.4 | O(18)-C(16)-C(17)-H(37) | -66.3 | H(41)-O(20)-C(26)-H(53) | 83.9 |
| C(2)-C(1)-N(9)-H(20) | -17.4 | H(25)-N(9)-Cu(14)-O(12) | 107.3 | N(9)-Co(14)-O(18)-C(16) | -7.3 | N(9)-Ni(14)-O(21)-C(24) | -172.4 | H(41)-O(20)-C(26)-H(54) | -160.6 |
| O(8)-C(1)-N(9)-N(10) | 11.1 | H(25)-N(9)-Cu(14)-O(18) | -74.4 | O(12)-Co(14)-O(18)-C(16) | 68.4 | N(9)-Ni(14)-O(21)-H(51) | -56.0 | Zn(14)-O(19)-C(22)-C(23) | -81.8 |
| O(8)-C(1)-N(9)-H(20) | 164.6 | H(25)-N(9)-Cu(14)-O(15) | -59.5 | O(19)-Co(14)-O(18)-C(16) | 152.0 | O(12)-Ni(14)-O(21)-C(24) | 57.3 | Zn(14)-O(19)-C(22)-H(43) | 39.1 |
| O(8)-C(1)-C(2)-C(3) | -146.6 | C(1)-N(9)-N(10)-C(11) | 136.5 | O(21)-Co(14)-O(18)-C(16) | -114.5 | O(12)-Ni(14)-O(21)-H(51) | 173.7 | Zn(14)-O(19)-C(22)-H(44) | 154.8 |
| O(8)-C(1)-C(2)-C(7) | 31.7 | H(25)-N(9)-N(10)-C(11) | -104.8 | O(20)-Co(14)-O(18)-C(16) | -144.7 | O(18)-Ni(14)-O(21)-C(24) | -89.7 | H(40)-O(19)-C(22)-C(23) | 63.1 |
| N(9)-C(1)-C(2)-C(3) | 35.3 | Cu(14)-N(9)-N(10)-C(11) | 7.8 | C(11)-C(13)-C(22)-N(23) | 19.7 | O(18)-Ni(14)-O(21)-H(51) | 26.7 | H(40)-O(19)-C(22)-H(43) | -175.9 |
| N(9)-C(1)-C(2)-C(7) | -146.3 | N(5)-C(6)-C(7)-C(2) | 1.5 | H(29)-C(13)-C(22)-N(23) | -101.4 | O(19)-Ni(14)-O(21)-C(24) | 8.8 | H(40)-O(19)-C(22)-H(44) | -60.3 |
|  |  | N(5)-C(6)-C(7)-H(24) | -178.6 | H(30)-C(13)-C(22)-N(23) | 139.5 | O(19)-Ni(14)-O(21)-H(51) | 125.1 | O(15)-C(16)-O(18)-Zn(14) | 4.8 |
|  |  | H(23)-C(6)-C(7)-C(2) | -179.0 | C(11)-O(12)-Co(14)-N(9) | -19.4 | O(20)-Ni(14)-O(21)-C(24) | 94.2 | C(17)-C(16)-O(18)-Zn(14) | -175.7 |
|  |  | H(23)-C(6)-C(7)-H(24) | 1.0 | C(11)-O(12)-Co(14)-O(18) | -118.8 | O(20)-Ni(14)-O(21)-H(51) | -149.4 | H(59)-C(16)-O(18)-Zn(14) | -1.3 |
|  |  | C(4)-N(5)-C(6)-C(7) | 0.0 | C(11)-O(12)-Co(14)-O(19) | 159.3 | N(9)-Ni(14)-O(20)-H(39) | -98.0 | O(15)-C(16)-C(17)-H(37) | 111.1 |
|  |  | C(4)-N(5)-C(6)-H(23) | -179.6 | C(11)-O(12)-Co(14)-O(21) | 119.4 | N(9)-Ni(14)-O(20)-H(40) | 14.8 | O(15)-C(16)-C(17)-H(38) | -131.6 |
|  |  | C(3)-C(4)-N(5)-C(6) | -1.2 | C(11)-O(12)-Co(14)-O(20) | 65.4 | O(12)-Ni(14)-O(20)-H(39) | -161.9 | O(15)-C(16)-C(17)-H(39) | -9.1 |
|  |  | H(22)-C(4)-N(5)-C(6) | 178.8 | N(10)-C(11)-C(13)-C(22) | -119.1 | O(12)-Ni(14)-O(20)-H(40) | -49.0 | O(18)-C(16)-C(17)-H(37) | -68.3 |
|  |  | C(2)-C(3)-C(4)-N(5) | 1.0 | N(10)-C(11)-C(13)-H(29) | 1.9 | O(18)-Ni(14)-O(20)-H(39) | -106.8 | O(18)-C(16)-C(17)-H(38) | 48.9 |
|  |  | C(2)-C(3)-C(4)-H(22) | -179.0 | N(10)-C(11)-C(13)-H(30) | 121.5 | O(18)-Ni(14)-O(20)-H(40) | 6.1 | O(18)-C(16)-C(17)-H(39) | 171.4 |
|  |  | H(21)-C(3)-C(4)-N(5) | 179.1 | O(12)-C(11)-C(13)-C(22) | 59.5 | O(19)-Ni(14)-O(20)-H(39) | 124.1 | H(59)-C(16)-C(17)-H(37) | 126.3 |
|  |  | H(21)-C(3)-C(4)-H(22) | -1.0 | O(12)-C(11)-C(13)-H(29) | -179.4 | O(19)-Ni(14)-O(20)-H(40) | -123.0 | H(59)-C(16)-C(17)-H(38) | -116.5 |
|  |  | C(1)-C(2)-C(7)-C(6) | -178.6 | O(12)-C(11)-C(13)-H(30) | -59.9 | O(21)-Ni(14)-O(20)-H(39) | 21.7 | H(59)-C(16)-C(17)-H(39) | 6.0 |
|  |  | C(1)-C(2)-C(7)-H(24) | 1.5 | N(10)-C(11)-O(12)-Co(14) | 16.9 | O(21)-Ni(14)-O(20)-H(40) | 134.5 | H(58)-O(15)-C(16)-C(17) | 180.0 |
|  |  | C(3)-C(2)-C(7)-C(6) | -1.7 | C(13)-C(11)-O(12)-Co(14) | -161.5 | N(9)-Ni(14)-O(19)-C(22) | -94.6 | H(58)-O(15)-C(16)-O(18) | -0.6 |
|  |  | C(3)-C(2)-C(7)-H(24) | 178.4 | N(9)-N(10)-C(11)-O(12) | 5.5 | N(9)-Ni(14)-O(19)-H(38) | 3.6 | H(58)-O(15)-C(16)-H(59) | 9.2 |
|  |  | C(1)-C(2)-C(3)-C(4) | 177.2 | N(9)-N(10)-C(11)-C(13) | -176.1 | O(12)-Ni(14)-O(19)-C(22) | -91.5 | N(9)-Zn(14)-O(19)-C(22) | 143.5 |
|  |  | C(1)-C(2)-C(3)-H(21) | -0.9 | C(1)-N(9)-Co(14)-O(12) | 143.3 | O(12)-Ni(14)-O(19)-H(38) | 6.7 | N(9)-Zn(14)-O(19)-H(40) | -0.2 |
|  |  | C(7)-C(2)-C(3)-C(4) | 0.5 | C(1)-N(9)-Co(14)-O(18) | -124.5 | O(18)-Ni(14)-O(19)-C(22) | -179.3 | O(12)-Zn(14)-O(19)-C(22) | 76.4 |
|  |  | C(7)-C(2)-C(3)-H(21) | -177.5 | C(1)-N(9)-Co(14)-O(19) | 139.4 | O(18)-Ni(14)-O(19)-H(38) | -81.1 | O(12)-Zn(14)-O(19)-H(40) | -67.3 |
|  |  | C(2)-C(1)-N(9)-N(10) | -45.4 | C(1)-N(9)-Co(14)-O(21) | -34.3 | O(20)-Ni(14)-O(19)-C(22) | -3.2 | O(18)-Zn(14)-O(19)-C(22) | -27.5 |
|  |  | C(2)-C(1)-N(9)-H(25) | -166.0 | C(1)-N(9)-Co(14)-O(20) | 50.3 | O(20)-Ni(14)-O(19)-H(38) | 95.0 | O(18)-Zn(14)-O(19)-H(40) | -171.3 |
|  |  | C(2)-C(1)-N(9)-Cu(14) | 81.8 | N(10)-N(9)-Co(14)-O(12) | 20.6 | O(21)-Ni(14)-O(19)-C(22) | 83.9 | O(20)-Zn(14)-O(19)-C(22) | -124.8 |
|  |  | O(8)-C(1)-N(9)-N(10) | 140.8 | N(10)-N(9)-Co(14)-O(18) | 112.8 | O(21)-Ni(14)-O(19)-H(38) | -178.0 | O(20)-Zn(14)-O(19)-H(40) | 91.5 |
|  |  | O(8)-C(1)-N(9)-H(25) | 20.1 | N(10)-N(9)-Co(14)-O(19) | 16.8 | N(9)-Ni(14)-O(18)-C(16) | 92.3 | O(21)-Zn(14)-O(19)-C(22) | 147.7 |
|  |  | O(8)-C(1)-N(9)-Cu(14) | -92.0 | N(10)-N(9)-Co(14)-O(21) | -157.0 | O(12)-Ni(14)-O(18)-C(16) | 156.2 | O(21)-Zn(14)-O(19)-H(40) | 3.9 |
|  |  | O(8)-C(1)-C(2)-C(3) | -141.5 | N(10)-N(9)-Co(14)-O(20) | -72.3 | O(19)-Ni(14)-O(18)-C(16) | -130.1 | N(9)-Zn(14)-O(21)-C(24) | 151.4 |
|  |  | O(8)-C(1)-C(2)-C(7) | 35.2 | H(28)-N(9)-Co(14)-O(12) | -94.2 | O(20)-Ni(14)-O(18)-C(16) | 101.1 | N(9)-Zn(14)-O(21)-H(42) | -88.7 |
|  |  | N(9)-C(1)-C(2)-C(3) | 45.0 | H(28)-N(9)-Co(14)-O(18) | -1.9 | O(21)-Ni(14)-O(18)-C(16) | -27.1 | O(12)-Zn(14)-O(21)-C(24) | -138.2 |
|  |  | N(9)-C(1)-C(2)-C(7) | -138.3 | H(28)-N(9)-Co(14)-O(19) | -98.0 | C(11)-C(13)-C(26)-N(27) | -138.2 | O(12)-Zn(14)-O(21)-H(42) | -18.3 |
|  |  |  |  | H(28)-N(9)-Co(14)-O(21) | 88.2 | H(33)-C(13)-C(26)-N(27) | 98.7 | O(18)-Zn(14)-O(21)-C(24) | -37.5 |
|  |  |  |  | H(28)-N(9)-Co(14)-O(20) | 172.9 | H(34)-C(13)-C(26)-N(27) | -18.9 | O(18)-Zn(14)-O(21)-H(42) | 82.4 |
|  |  |  |  | C(1)-N(9)-N(10)-C(11) | -129.9 | C(11)-O(12)-Ni(14)-N(9) | 19.9 | O(20)-Zn(14)-O(21)-C(24) | 58.1 |
|  |  |  |  | H(28)-N(9)-N(10)-C(11) | 77.4 | C(11)-O(12)-Ni(14)-O(18) | -60.3 | O(20)-Zn(14)-O(21)-H(42) | 178.0 |
|  |  |  |  | Co(14)-N(9)-N(10)-C(11) | -18.5 | C(11)-O(12)-Ni(14)-O(19) | -157.7 | O(19)-Zn(14)-O(21)-C(24) | 147.3 |
|  |  |  |  | N(5)-C(6)-C(7)-C(2) | -0.2 | C(11)-O(12)-Ni(14)-O(20) | 115.6 | O(19)-Zn(14)-O(21)-H(42) | -92.8 |
|  |  |  |  | N(5)-C(6)-C(7)-H(27) | -179.2 | C(11)-O(12)-Ni(14)-O(21) | 152.5 | N(9)-Zn(14)-O(20)-C(26) | 81.3 |
|  |  |  |  | H(26)-C(6)-C(7)-C(2) | 179.8 | N(10)-C(11)-C(13)-C(26) | -142.9 | N(9)-Zn(14)-O(20)-H(41) | -158.4 |
|  |  |  |  | H(26)-C(6)-C(7)-H(27) | 0.7 | N(10)-C(11)-C(13)-H(33) | -19.8 | O(12)-Zn(14)-O(20)-C(26) | 116.3 |
|  |  |  |  | C(4)-N(5)-C(6)-C(7) | 0.5 | N(10)-C(11)-C(13)-H(34) | 97.8 | O(12)-Zn(14)-O(20)-H(41) | -123.4 |
|  |  |  |  | C(4)-N(5)-C(6)-H(26) | -179.4 | O(12)-C(11)-C(13)-C(26) | 38.4 | O(18)-Zn(14)-O(20)-C(26) | -99.8 |
|  |  |  |  | C(3)-C(4)-N(5)-C(6) | -0.3 | O(12)-C(11)-C(13)-H(33) | 161.6 | O(18)-Zn(14)-O(20)-H(41) | 20.6 |
|  |  |  |  | H(25)-C(4)-N(5)-C(6) | 179.5 | O(12)-C(11)-C(13)-H(34) | -80.8 | O(21)-Zn(14)-O(20)-C(26) | 159.9 |
|  |  |  |  | C(2)-C(3)-C(4)-N(5) | -0.2 | N(10)-C(11)-O(12)-Ni(14) | -24.6 | O(21)-Zn(14)-O(20)-H(41) | -79.7 |
|  |  |  |  | C(2)-C(3)-C(4)-H(25) | 180.0 | C(13)-C(11)-O(12)-Ni(14) | 153.7 | O(19)-Zn(14)-O(20)-C(26) | 1.0 |
|  |  |  |  | H(24)-C(3)-C(4)-N(5) | -179.9 | N(9)-N(10)-C(11)-O(12) | 0.0 | O(19)-Zn(14)-O(20)-H(41) | 121.4 |
|  |  |  |  | H(24)-C(3)-C(4)-H(25) | 0.3 | N(9)-N(10)-C(11)-C(13) | -178.4 | N(9)-Zn(14)-O(18)-C(16) | 150.9 |
|  |  |  |  | C(1)-C(2)-C(7)-C(6) | -179.8 | C(1)-N(9)-Ni(14)-O(12) | 108.6 | O(12)-Zn(14)-O(18)-C(16) | 143.7 |
|  |  |  |  | C(1)-C(2)-C(7)-H(27) | -0.7 | C(1)-N(9)-Ni(14)-O(18) | -157.2 | O(20)-Zn(14)-O(18)-C(16) | -22.4 |
|  |  |  |  | C(3)-C(2)-C(7)-C(6) | -0.4 | C(1)-N(9)-Ni(14)-O(19) | 111.9 | O(21)-Zn(14)-O(18)-C(16) | 64.1 |
|  |  |  |  | C(3)-C(2)-C(7)-H(27) | 178.7 | C(1)-N(9)-Ni(14)-O(20) | 23.5 | O(19)-Zn(14)-O(18)-C(16) | -117.6 |
|  |  |  |  | C(1)-C(2)-C(3)-C(4) | 179.9 | C(1)-N(9)-Ni(14)-O(21) | -66.4 | C(11)-C(13)-C(28)-N(29) | -62.9 |
|  |  |  |  | C(1)-C(2)-C(3)-H(24) | -0.4 | N(10)-N(9)-Ni(14)-O(12) | -15.9 | H(35)-C(13)-C(28)-N(29) | 58.6 |
|  |  |  |  | C(7)-C(2)-C(3)-C(4) | 0.6 | N(10)-N(9)-Ni(14)-O(18) | 78.3 | H(36)-C(13)-C(28)-N(29) | 177.4 |
|  |  |  |  | C(7)-C(2)-C(3)-H(24) | -179.7 | N(10)-N(9)-Ni(14)-O(19) | -12.5 | C(11)-O(12)-Zn(14)-N(9) | -20.9 |
|  |  |  |  | C(2)-C(1)-N(9)-N(10) | -153.8 | N(10)-N(9)-Ni(14)-O(20) | -101.0 | C(11)-O(12)-Zn(14)-O(18) | 158.0 |
|  |  |  |  | C(2)-C(1)-N(9)-H(28) | -3.2 | N(10)-N(9)-Ni(14)-O(21) | 169.2 | C(11)-O(12)-Zn(14)-O(20) | -58.9 |
|  |  |  |  | C(2)-C(1)-N(9)-Co(14) | 95.7 | H(32)-N(9)-Ni(14)-O(12) | -133.8 | C(11)-O(12)-Zn(14)-O(21) | -103.6 |
|  |  |  |  | O(8)-C(1)-N(9)-N(10) | 30.9 | H(32)-N(9)-Ni(14)-O(18) | -39.7 | C(11)-O(12)-Zn(14)-O(19) | 56.0 |
|  |  |  |  | O(8)-C(1)-N(9)-H(28) | -178.5 | H(32)-N(9)-Ni(14)-O(19) | -130.5 | N(10)-C(11)-C(13)-C(28) | 116.2 |
|  |  |  |  | O(8)-C(1)-N(9)-Co(14) | -79.6 | H(32)-N(9)-Ni(14)-O(20) | 141.1 | N(10)-C(11)-C(13)-H(35) | -4.9 |
|  |  |  |  | O(8)-C(1)-C(2)-C(3) | -174.7 | H(32)-N(9)-Ni(14)-O(21) | 51.2 | N(10)-C(11)-C(13)-H(36) | -124.4 |
|  |  |  |  | O(8)-C(1)-C(2)-C(7) | 4.7 | C(1)-N(9)-N(10)-C(11) | -72.1 | O(12)-C(11)-C(13)-C(28) | -63.4 |
|  |  |  |  | N(9)-C(1)-C(2)-C(3) | 10.0 | Ni(14)-N(9)-N(10)-C(11) | 11.2 | O(12)-C(11)-C(13)-H(35) | 175.4 |
|  |  |  |  | N(9)-C(1)-C(2)-C(7) | -170.7 | H(32)-N(9)-N(10)-C(11) | 139.1 | O(12)-C(11)-C(13)-H(36) | 55.9 |
|  |  |  |  |  |  | N(5)-C(6)-C(7)-C(2) | -1.1 | N(10)-C(11)-O(12)-Zn(14) | 19.9 |
|  |  |  |  |  |  | N(5)-C(6)-C(7)-H(31) | 179.1 | C(13)-C(11)-O(12)-Zn(14) | -160.5 |
|  |  |  |  |  |  | H(30)-C(6)-C(7)-C(2) | 179.1 | N(9)-N(10)-C(11)-O(12) | 5.4 |
|  |  |  |  |  |  | H(30)-C(6)-C(7)-H(31) | -0.7 | N(9)-N(10)-C(11)-C(13) | -174.2 |
|  |  |  |  |  |  | C(4)-N(5)-C(6)-C(7) | -0.1 | C(1)-N(9)-Zn(14)-O(12) | 144.5 |
|  |  |  |  |  |  | C(4)-N(5)-C(6)-H(30) | 179.8 | C(1)-N(9)-Zn(14)-O(18) | 137.0 |
|  |  |  |  |  |  | C(3)-C(4)-N(5)-C(6) | 0.8 | C(1)-N(9)-Zn(14)-O(20) | -49.7 |
|  |  |  |  |  |  | H(29)-C(4)-N(5)-C(6) | -178.7 | C(1)-N(9)-Zn(14)-O(21) | -134.6 |
|  |  |  |  |  |  | C(2)-C(3)-C(4)-N(5) | -0.3 | C(1)-N(9)-Zn(14)-O(19) | 43.9 |
|  |  |  |  |  |  | C(2)-C(3)-C(4)-H(29) | 179.2 | N(10)-N(9)-Zn(14)-O(12) | 20.9 |
|  |  |  |  |  |  | H(28)-C(3)-C(4)-N(5) | -177.6 | N(10)-N(9)-Zn(14)-O(18) | 13.4 |
|  |  |  |  |  |  | H(28)-C(3)-C(4)-H(29) | 1.9 | N(10)-N(9)-Zn(14)-O(20) | -173.3 |
|  |  |  |  |  |  | C(1)-C(2)-C(7)-C(6) | 179.5 | N(10)-N(9)-Zn(14)-O(21) | 101.9 |
|  |  |  |  |  |  | C(1)-C(2)-C(7)-H(31) | -0.7 | N(10)-N(9)-Zn(14)-O(19) | -79.6 |
|  |  |  |  |  |  | C(3)-C(2)-C(7)-C(6) | 1.5 | H(34)-N(9)-Zn(14)-O(12) | -93.8 |
|  |  |  |  |  |  | C(3)-C(2)-C(7)-H(31) | -178.7 | H(34)-N(9)-Zn(14)-O(18) | -101.3 |
|  |  |  |  |  |  | C(1)-C(2)-C(3)-C(4) | -178.8 | H(34)-N(9)-Zn(14)-O(20) | 72.0 |
|  |  |  |  |  |  | C(1)-C(2)-C(3)-H(28) | -1.5 | H(34)-N(9)-Zn(14)-O(21) | -12.8 |
|  |  |  |  |  |  | C(7)-C(2)-C(3)-C(4) | -0.9 | H(34)-N(9)-Zn(14)-O(19) | 165.7 |
|  |  |  |  |  |  | C(7)-C(2)-C(3)-H(28) | 176.4 | C(1)-N(9)-N(10)-C(11) | -136.1 |
|  |  |  |  |  |  | C(2)-C(1)-N(9)-N(10) | -165.4 | H(34)-N(9)-N(10)-C(11) | 88.2 |
|  |  |  |  |  |  | C(2)-C(1)-N(9)-Ni(14) | 106.8 | Zn(14)-N(9)-N(10)-C(11) | -18.8 |
|  |  |  |  |  |  | C(2)-C(1)-N(9)-H(32) | -17.9 | N(5)-C(6)-C(7)-C(2) | -0.6 |
|  |  |  |  |  |  | O(8)-C(1)-N(9)-N(10) | 16.1 | N(5)-C(6)-C(7)-H(33) | -177.6 |
|  |  |  |  |  |  | O(8)-C(1)-N(9)-Ni(14) | -71.7 | H(32)-C(6)-C(7)-C(2) | 179.2 |
|  |  |  |  |  |  | O(8)-C(1)-N(9)-H(32) | 163.6 | H(32)-C(6)-C(7)-H(33) | 2.2 |
|  |  |  |  |  |  | O(8)-C(1)-C(2)-C(3) | 144.0 | C(4)-N(5)-C(6)-C(7) | 0.7 |
|  |  |  |  |  |  | O(8)-C(1)-C(2)-C(7) | -33.9 | C(4)-N(5)-C(6)-H(32) | -179.0 |
|  |  |  |  |  |  | N(9)-C(1)-C(2)-C(3) | -34.6 | C(3)-C(4)-N(5)-C(6) | 0.1 |
|  |  |  |  |  |  | N(9)-C(1)-C(2)-C(7) | 147.6 | H(31)-C(4)-N(5)-C(6) | 179.9 |
|  |  |  |  |  |  |  |  | C(2)-C(3)-C(4)-N(5) | -1.0 |
|  |  |  |  |  |  |  |  | C(2)-C(3)-C(4)-H(31) | 179.2 |
|  |  |  |  |  |  |  |  | H(30)-C(3)-C(4)-N(5) | 179.4 |
|  |  |  |  |  |  |  |  | H(30)-C(3)-C(4)-H(31) | -0.5 |
|  |  |  |  |  |  |  |  | C(1)-C(2)-C(7)-C(6) | 179.7 |
|  |  |  |  |  |  |  |  | C(1)-C(2)-C(7)-H(33) | -3.4 |
|  |  |  |  |  |  |  |  | C(3)-C(2)-C(7)-C(6) | -0.3 |
|  |  |  |  |  |  |  |  | C(3)-C(2)-C(7)-H(33) | 176.6 |
|  |  |  |  |  |  |  |  | C(1)-C(2)-C(3)-C(4) | -179.0 |
|  |  |  |  |  |  |  |  | C(1)-C(2)-C(3)-H(30) | 0.7 |
|  |  |  |  |  |  |  |  | C(7)-C(2)-C(3)-C(4) | 1.1 |
|  |  |  |  |  |  |  |  | C(7)-C(2)-C(3)-H(30) | -179.3 |
|  |  |  |  |  |  |  |  | C(2)-C(1)-N(9)-N(10) | -145.2 |
|  |  |  |  |  |  |  |  | C(2)-C(1)-N(9)-H(34) | -11.2 |
|  |  |  |  |  |  |  |  | C(2)-C(1)-N(9)-Zn(14) | 101.5 |
|  |  |  |  |  |  |  |  | O(8)-C(1)-N(9)-N(10) | 36.6 |
|  |  |  |  |  |  |  |  | O(8)-C(1)-N(9)-H(34) | 170.6 |
|  |  |  |  |  |  |  |  | O(8)-C(1)-N(9)-Zn(14) | -76.7 |
|  |  |  |  |  |  |  |  | O(8)-C(1)-C(2)-C(3) | -22.0 |
|  |  |  |  |  |  |  |  | O(8)-C(1)-C(2)-C(7) | 157.9 |
|  |  |  |  |  |  |  |  | N(9)-C(1)-C(2)-C(3) | 159.7 |
|  |  |  |  |  |  |  |  | N(9)-C(1)-C(2)-C(7) | -20.3 |

Table S5. Comparison of ***In vitro* Cytotoxicity IC_50_ (µM)** values of synthesized ligand and its complexes with previously reported anticancer agents and analogues compounds

| **Compound** | ***In vitro* Cytotoxicity IC_50_ (µM)^*^** | | ***Reference No.*** |
| --- | --- | --- | --- |
|  | **HepG2** | **HCT-116** |  |
| **H_2_L** | 3.87±0.2 (strong) | 4.93±0.3 (strong) | Current work |
| **Cu(II)** | 53.24±3.0 (weak) | 59.20±3.2 (weak) | Current work |
| **Co(II)** | 67.29±3.7 (weak) | 74.12±3.9 (weak) | Current work |
| **Ni(II)** | 58.31±3.3 (weak) | 65.24±3.6 (weak) | Current work |
| **Zn(II)** | >100 (non-cytotoxic) | >100 (non-cytotoxic) | Current work |
| **DOX^**^** | 4.50±0.2 (strong) | 5.23±0.3 (strong) | Current work |
| Fluorouracil (5-FU) | 12.92±0.085 (strong) | 26.98±1.87 (moderate) | [72] |
| Cis-platin | 6.50±0.38 (very strong) | 8.51±0.25 (very strong) | [73] |
| INHPHC | 10.62±1.0 (strong) | 23.14±1.6 (moderate) | [22] |
| Zn(II)-INHPHC | 61.03±3.3 (weak) | 73.76±3.5 (weak) | [22] |
| NBINHC | 13.71±1.2 (strong) | 17.64±1.4 (moderate) | [23] |
| Zn(II)-NBINHC | 55.36±2.9 (weak | 45.29±2.5 (moderate) | [23] |
| INHCTB | 8.90±0.8 (very strong) | 11.32±1.1 (strong) | [24] |
| Zn(II)-INHCTB | 26.20±1.7 (moderate) | 34.60±2.0 (moderate) | [24] |
| INHNPC | 8.44±0.9 (very strong) | 7.68±0.8 (very strong) | [25] |
| Zn(II)-INHNPC | 38.30±2.4 (moderate) | 53.00±3.1 (weak) | [25] |
| CNNH | 6.59±0.4 (very strong) | 8.17±0.7 (very strong) | [27] |
| Cu(II)-CNNH | 72.35±3.8 (weak) | 84.13±4.3 (weak) | [27] |
| Co(II)-CNNH | 49.22±2.9 (moderate) | 79.55±4.1 (weak) | [27] |
| Ni(II)-CNNH | 45.72±2.7 (moderate) | 95.13±5.2 (weak) | [27] |
| Zn(II)-CNNH | 28.34±2.1 (moderate) | 43.84±2.5 (moderate) | [27] |
| HBPHC | 8.53±0.6 (very strong) | 7.87±0.5 very strong | [28] |
| Cu(II)-HBPHC | 23.10±1.8 (moderate) | 31.51±2.2 (moderate) | [28] |
| Co(II)-HBPHC | 42.77±2.6 (moderate) | 59.72±3.4 (weak) | [28] |
| Ni(II)-HBPHC | 11.89±0.9 (strong) | 13.78±1.0 (strong) | [28] |
| Zn(II)-HBPHC | 29.80±2.2 (moderate) | 38.11±2.5 (moderate) | [28] |
| FMHBH | 30.72±2.2 (moderate) | 35.40±2.2 (moderate) | [29] |
| Cu(II)-FMHBH | 82.63±4.2 (weak) | >100 (non-cytotoxic) | [29] |
| Co(II)-FMHBH | 93.56±4.7 (weak) | 89.01±4.5 (weak) | [29] |
| Ni(II)-FMHBH | 41.47±2.6 (moderate) | 39.19±2.4 (moderate) | [29] |
| Zn(II)-FMHBH | 32.83±2.3 (moderate) | 37.27±2.4 (moderate) | [29] |

* IC50 (µM): 1-10 (very strong), 11-20 (strong), 21-50 (moderate), 51-100 (weak) and above 100 (non-cytotoxic).

** DOX = Doxorubicin

INHCTB [24] *N*-(2-isonicotinoylhydrazine-carbonothioyl)benzamide

INHPHC [22] 2-isonicotinoyl-N-phenylhydrazine-1-carboxamide

INHNPC [25] 2‐isonicotinoyl‐N‐phenylhydrazine‐1‐carbothioamide

NBINHC [23] N-benzyl-2-isonicotinoylhydrazine-1-carbothioamide

FMHBH [29] *N’*-(Furan-2-ylmethylene)-2-hydroxybenzohydrazide

CNNH [27] N’-(2-cyanoacetyl)nicotinohydrazide

HBPHC [28] 2-(2-hydroxybenzoyl)-N-phenylhydrazine-1-carbothioamide
